# Supplementary material for: An Early Miocene kentriodontoid (Cetacea: Odontoceti) from the western North Pacific, and its implications for their phylogeny and paleobiogeography
Source: PLoS One. 2023 Feb 15;18(2):e0280218. doi: 10.1371/journal.pone.0280218 (PMC9931143; doi:10.1371/journal.pone.0280218)
Supplement: Attachment — Submitted filename: PONE-D-21-33588 (1)_JG.pdf [file pone.0280218.s006.pdf]

PLOS ONE

An early Miocene kentriodontoid (Cetacea: Odontoceti) from the western North Pacific, and its implications for their phylogeny and paleobiogeography

--Manuscript Draft--

**Manuscript Number:**	PONE-D-21-33588
**Article Type:**	Research Article
**Full Title:**	An early Miocene kentriodontoid (Cetacea: Odontoceti) from the western North Pacific, and its implications for their phylogeny and paleobiogeography
**Short Title:**	Phylogeny and palaeobiogeography of kentriodontoids
**Corresponding Author:**	Zixuan Guo, M.D.<br>University of Tsukuba: Tsukuba Daigaku<br>Tsukuba, Ibaraki JAPAN
**Keywords:**	Odontoceti; kentriodontid; Japan; Miocene; Phylogeny; palaeobiogeography
**Abstract:**	So–called 'kentriodontids' are extinct dolphin–like odontocetes known from the early to late Miocene worldwide. Although recent studies have proposed that they are monophyletic, their taxonomic relationships remain controversial. Such a controversy exists partly because of the predominance of primitive morphologies in this taxon, but the fact is that quite a few 'kentriodontids' are known only from fragmentary skulls and/or isolated periotics. A new 'kentriodontid' Platysvercus ugonis gen. et sp. nov. is described based on a nearly complete skull from the upper lower Miocene Sugota Formation , Akita Prefecture, northern Japan. Based on the phylogenetic analysis of P . ugonis described here, the monophyly of the 'kentriodontids' is confirmed, and it could be recognized as the superfamily Kentriodontoidea because of its robustness. This new superfamily is subdivided into two families: Kentriodontidae and Delphinodontidae fam. nov. Based on the paleobiogeographic analysis of Kentriodontoidea, their common ancestor emerged in the North Pacific Ocean and spread over the Northern Hemisphere. Initial diversification of the Kentriodontidae in the North Pacific Ocean and Delphinodontidae in the North Atlantic Ocean was recognized as a vicariance event. The vicissitudes of Kentriodontoidea could have been synchronously influenced by climate events during the middle Miocene.
**Order of Authors:**	Zixuan Guo, M.D.
	Naoki Kohno
**Additional Information:**	
**Question**	**Response**
**Financial Disclosure**<br><br>Enter a financial disclosure statement that describes the sources of funding for the work included in this submission. Review the submission guidelines for detailed requirements. View published research articles from *PLOS ONE* for specific examples.<br><br>This statement is required for submission and **will appear in the published article** if the submission is accepted. Please make sure it is accurate.	The authors received no specific funding for this work.

Powered by Editorial Manager® and ProduXion Manager® from Aries Systems Corporation

**Unfunded studies**<br>Enter: *The author(s) received no specific funding for this work.*<br><br>**Funded studies**<br>Enter a statement with the following details:<br>• Initials of the authors who received each award<br>• Grant numbers awarded to each author<br>• The full name of each funder<br>• URL of each funder website<br>• Did the sponsors or funders play any role in the study design, data collection and analysis, decision to publish, or preparation of the manuscript?<br>• **NO** - Include this sentence at the end of your statement: *The funders had no role in study design, data collection and analysis, decision to publish, or preparation of the manuscript.*<br>• **YES** - Specify the role(s) played.<br><br>* typeset	
**Competing Interests**<br><br>Use the instructions below to enter a competing interest statement for this submission. On behalf of all authors, disclose any competing interests that could be perceived to bias this work—acknowledging all financial support and any other relevant financial or non-financial competing interests.<br><br>This statement is required for submission and **will appear in the published article** if the submission is accepted. Please make sure it is accurate and that any funding sources listed in your Funding Information later in the submission form are also declared in your Financial Disclosure statement.<br><br>View published research articles from *PLOS ONE* for specific examples.	The authors have declared that no competing interests exist.

**NO authors have competing interests**<br><br>Enter: *The authors have declared that no competing interests exist.*<br><br>**Authors with competing interests**<br><br>Enter competing interest details beginning with this statement:<br><br>*I have read the journal's policy and the authors of this manuscript have the following competing interests: [insert competing interests here]*<br><br>* typeset	
**Ethics Statement**<br><br>Enter an ethics statement for this submission. This statement is required if the study involved:<br><br>• Human participants<br>• Human specimens or tissue<br>• Vertebrate animals or cephalopods<br>• Vertebrate embryos or tissues<br>• Field research<br><br>Write "N/A" if the submission does not require an ethics statement.<br><br>General guidance is provided below. Consult the submission guidelines for detailed instructions. **Make sure that all information entered here is included in the Methods section of the manuscript.**	N/A

**Format for specific study types**<br><br>**Human Subject Research (involving human participants and/or tissue)**<br>• Give the name of the institutional review board or ethics committee that approved the study<br>• Include the approval number and/or a statement indicating approval of this research<br>• Indicate the form of consent obtained (written/oral) or the reason that consent was not obtained (e.g. the data were analyzed anonymously)<br><br>**Animal Research (involving vertebrate animals, embryos or tissues)**<br>• Provide the name of the Institutional Animal Care and Use Committee (IACUC) or other relevant ethics board that reviewed the study protocol, and indicate whether they approved this research or granted a formal waiver of ethical approval<br>• Include an approval number if one was obtained<br>• If the study involved *non-human primates*, add *additional details* about animal welfare and steps taken to ameliorate suffering<br>• If anesthesia, euthanasia, or any kind of animal sacrifice is part of the study, include briefly which substances and/or methods were applied<br><br>**Field Research**<br><br>Include the following details if this study involves the collection of plant, animal, or other materials from a natural setting:<br>• Field permit number<br>• Name of the institution or relevant body that granted permission	
**Data Availability**<br><br>Authors are required to make all data underlying the findings described fully available, without restriction, and from the time of publication. PLOS allows rare exceptions to address legal and ethical concerns. See the PLOS Data Policy and FAQ for detailed information.	Yes - all data are fully available without restriction

A Data Availability Statement describing where the data can be found is required at submission. Your answers to this question constitute the Data Availability Statement and **will be published in the article**, if accepted.<br><br>**Important:** Stating 'data available on request from the author' is not sufficient. If your data are only available upon request, select 'No' for the first question and explain your exceptional situation in the text box.<br><br>Do the authors confirm that all data underlying the findings described in their manuscript are fully available without restriction?	
**Describe where the data may be found in full sentences. If you are copying our sample text, replace any instances of XXX with the appropriate details.**<br><br>• If the data are **held or will be held in a public repository**, include URLs, accession numbers or DOIs. If this information will only be available after acceptance, indicate this by ticking the box below. For example: *All XXX files are available from the XXX database (accession number(s) XXX, XXX.).*<br>• If the data are all contained **within the manuscript and/or Supporting Information files**, enter the following: *All relevant data are within the manuscript and its Supporting Information files.*<br>• If neither of these applies but you are able to provide **details of access elsewhere**, with or without limitations, please do so. For example:<br><br>*Data cannot be shared publicly because of [XXX]. Data are available from the XXX Institutional Data Access / Ethics Committee (contact via XXX) for researchers who meet the criteria for access to confidential data.*<br><br>*The data underlying the results presented in the study are available from (include the name of the third party*	All relevant data are within the manuscript and its Supporting Information files.

and contact information or URL).<br>• This text is appropriate if the data are owned by a third party and authors do not have permission to share the data.<br><br>* typeset	
Additional data availability information:	

Manuscript
Click here to access/download;Manuscript;Guo&KohnoManuscript_1020clear.

An early Miocene kentriodontoid (Cetacea: Odontoceti) from the western North Pacific, and its implications for their phylogeny and paleobiogeography

Zixuan Guo[1*], Naoki Kohno[1,2]

[1] Graduate School of Life and Environmental Sciences, University of Tsukuba, Tsukuba, Japan

[2] Department of Geology and Paleontology, National Museum of Nature and Science, Tsukuba, Japan

* Corresponding author

E-mail: guo_z@geol.tsukuba.ac.jp

Abstract

So–called ‘kentriodontids’ are extinct dolphin–like odontocetes known from the early to late Miocene worldwide. Although recent studies have proposed that they are monophyletic, their taxonomic relationships remain controversial. Such a controversy exists partly because of the predominance of primitive morphologies in this taxon, but the fact is that quite a few ‘kentriodontids’ are known only from fragmentary skulls and/or isolated periotics. A new ‘kentriodontid’ *Platysvercus ugonis* gen. et sp. nov. is described based on a nearly complete skull from the upper lower Miocene Sugota Formation, Akita Prefecture, northern Japan. Based on the phylogenetic analysis of *P. ugonis* described here, the monophyly of the ‘kentriodontids’ is confirmed, and it could be recognized as the superfamily Kentriodontoidea because of its robustness. This new superfamily is subdivided into two families: Kentriodontidae and Delphinodontidae fam. nov. Based on the paleobiogeographic analysis of Kentriodontoidea, their common ancestor emerged in the North Pacific Ocean and spread over the Northern Hemisphere. Initial diversification of the Kentriodontidae in the North Pacific Ocean and Delphinodontidae in the North Atlantic Ocean was recognized as a vicariance event. The vicissitudes of Kentriodontoidea could have been synchronously influenced by climate events during the middle Miocene.

Introduction

So-called ‘Kentriodontids’ are an extinct group of odontocetes known from the early to late Miocene worldwide. At least 14 genera and 26 species are described as ‘kentriodontids,’ and they are sometimes recognized as extinct stem taxa within the Delphinida, including the Delphinoidea and Inioidea [1–5]. Although many studies have concentrated on understanding their inter–generic and inter–specific relationships within the Delphinida, quite a few described species of ‘kentriodontids’ are so fragmentary and they have sometimes been classified as paraphyletic and/or unresolved taxa. There needs to be a review of ~~their whole aspect~~ once more, because many new species have been reported in the past decades. Some recent studies [5–8] attempted to solve this problem. In particular, some of these studies [8,9] supported the monophyletic relationships of all previously known ‘kentriodontids’, which comprise the sister group of the Dephinoidea. However, further confirmation is needed for phylogenetic analysis because the topologies from recent studies on ‘kentriodontids’ were quite different among them [5–8]. Therefore, a more comprehensive study for ‘kentriodontids,’ especially their phylogenetic relationships, is necessary to refine the systematics of ‘kentriodontids.’

In addition to the phylogenetic relationships, previous studies on ‘kentriodontids’ suggested that their ecological niche was thought to be similar to the extant delphinoids [10,11]. ~~Their distribution and diversification have also been discussed by some early~~

~~studies (e.g. [4]); that is,~~ they show high diversity through the late early and early late Miocene, and their highly ~~diversified situation~~ during those periods is thought to be parallel to the present ~~time~~ Delphinoidea. In particular, the Delphinoidea ~~erupted~~ from the late Miocene [12,13], and this turnover event also remains unexplained. In addition, many geological events occurred during the Miocene, with notable changes ~~such as~~ climate change (for example, the Miocene Climatic Optimum and middle Miocene Climate Transition [14], tectonics [15], ocean circulation [16], and ice–sheet ~~fluctuation~~ [17]). However, only a few studies have investigated the effects of these events on marine vertebrates [18]. Accordingly, their geologic ages and ~~locality information~~ are important to understand the pattern and process of the evolution and diversification of ‘kentriodontids.’ Therefore, dense taxon sampling is necessary because, for instance, only ~~the~~ two ‘kentriodontid’ species [7,19] have been known from the western North Pacific.

Here we describe a new ‘kentriodontid’ skull from the upper lower Miocene of Japan as an addition to the western North Pacific record of ‘kentriodontids’ (Fig 1). Also, we locate its phylogenetic position and re-examine ‘kentriodontid’ phylogeny with further species added. We then try to ~~establish~~ their systematics and reconstruct their paleobiogeography based on our rigorous analysis, considering the evolution and diversification of ‘kentriodontids'.

Fig 1. Geographical and geological contexts of the type locality of *Platysvercus ugonis* gen. et sp. nov. (A) the type locality of *Platysvercus ugonis* gen. et sp. nov., holotype, UTHFM 00034. (B) planktonic foraminiferal zone and stratigraphic diagram, modified from Ozawa [40] and Wade et al. [39].

Materials and methods

Phylogenetic analysis

The phylogenetic position of the new specimen (i.e. UTHFM 00034) was analyzed using ~~a~~ data matrix of Guo and Kohno [8], which was modified in minor ways (see Supporting Information). In addition to the ‘kentriodontid’ taxa that were used for the phylogenetic analysis in Guo and Kohno [8], three previously known ‘kentriodontids’ (i.e., *Kentriodon hobetsu* from the middle Miocene of Japan, *Kentriodon hoepfneri* from the middle/upper Miocene of Germany and *Sophianacetus commenticius* from the middle Miocene of Hungary) were included in our analysis with UTHFM 00034. The character matrix and OTUs ~~originally prepared~~ by Guo and Kohno [8] are a combination of Tanaka et al. [20] and Kimura and Hasegawa [7], which were derived from those prepared by Peredo et al. [5], Lambert et al. [6], Tanaka and Fordyce [21], Geisler et al. [22], Murakami

et al. [12], and Geisler et al. [23]. This data matrix ~~is included~~ almost all ‘kentriodontids’ that are known at least by crania. The analysis performed here was based on 107 taxa and 387 morphological characters, with a backbone constraint ~~from extant~~ cetaceans [24–26] (see Supporting Information). In total, we included 21 previously known ‘kentriodontid’ taxa with UTHFM 00034 into our phylogenetic analysis (see Supporting Information).

Phylogenetic analysis was conducted with TNT 1.5 [27,28], using the ‘New Technology Search’ with default values, found minimum length trees 1,000 times with a backbone constraint from extant taxa [24–26], as mentioned above. All characters were treated as unweighted and unordered. To measure the stability of each node, Bremer support and bootstrap values were calculated for each node of the consensus tre[illegible]

Ancestral range reconstruction analysis

~~The~~ ancestral range reconstructions using the Statistical Dispersal–Vicariance Analysis (S–DIVA) [29], implemented in the software RASP 4 [30,31], ~~was~~ performed to ~~reconstruct~~ the paleobiogeographic distributions of ‘kentriodontids.’ The S–DIVA is a parsimony–based method to reconstruct the frequency of alternative ancestral distribution at each node in the phylogeny. S–DIVA considers vicariance events as carrying a lower cost than dispersal and extinction events, and then accounts for all possible ancestral distributions and calculates the probabilities.

All taxa within the most parsimonious trees were used in this analysis. The condensed tree was used by the user–specified consensus tree; i.e., the consensus tree from the phylogenetic analysis. Due to the distribution of marine mammals that are usually considerably broader than terrestrial animals with ambiguous distribution boundaries, we assigned seven distributional regions of the oceans as follows: (A) North Pacific Ocean, (B) North Atlantic Ocean, (C) South Pacific Ocean, (D) South Atlantic Ocean, (E) Indian Ocean, (F) Arctic Ocean, and (G) Southern Ocean, for both extinct and extant species of odontocetes. The distributional information for extant taxa was collected from the IUCN Red List of Threatened Species [32], and the extinct taxa were assigned based on the Paleobiology Database (PBDB, www.paleobiodb.org). The S–DIVA analysis was calculated with the following parameters: max areas at each node = 4; allow reconstruction with max reconstructions = 100; max reconstructions for final tree = 1000.

Institutional abbreviations

UTHFM, Ugo Town History and Folklore Museum, Ogachi County, Akita, Japan; NMNS–PV, fossil vertebrate collections at the Department of Geology and Paleontology, National Museum of Nature and Science, Tsukuba, Japan; NSMT–M, Marine Mammal Collection at the Department of Zoology, the National Museum of Nature and Science, Tsukuba, Japan.

Anatomical terminology

The skull terminology follows Mead and Fordyce [33] and Ichishima [34].

Systematic Paleontology

ARTIODACTYLA Owen, 1848

CETACEA Brisson, 1762

ODONTOCETI Flower, 1867

DELPHINIDA Muizon, 1984

Superfamily KENTRIODONTOIDEA (Slijper, 1936) new rank

Diagnosis of superfamily

It differs from other superfamilies of the Delphinida in having the following derived characters: the premaxillae at anterior of the rostrum mediolaterally compressed (character 3), the mesorostral groove posteriorly constricted, anteriorly abruptly diverges at the level anterior to the nares and behind the level of the antorbital notch (character 7), the lacrimal or the jugal forms the dorsolateral edge of internal opening of the infraorbital foramen (character 56), the infratemporal crest forms a curved ridge (character 62), the dorsal margin of the mesethmoid low (character 104), the alisphenoid laterally exposed broadly in the temporal fossa (character 157), the basioccipital crests angle 15°–40° in the ventral view (character 225), the hypoglossal foramen separated by a thick bone from the jugular foramen (character 227), the pars cochlearis ventrolaterally convex (character 278), the basihyal fused with the thyrohyal (character 326), and the transverse processes of lumbar vertebrae angle 45° or more (character 328).

Type family

Kentriodontidae Slijper, 1936.

Included families.

Kentriodontidae Slijper, 1936 and Delphinodontidae, fam. nov.

Etymology of superfamily

The superfamily name is from the type family, Kentriodontidae.

Family DELPHINODONTIDAE fam. nov.

LSID. urn:lsid:zoobank.org:act:FDDCC940-9068-4724-9374-8FD50FCFABB3

Diagnosis of family

It differs from the Kentriodontidae in having the following derived characters: 24–27 mandibular teeth (character 37), the premaxillary foramen located medially (character 70), a distinctly inflated process of the nasal at its anterolateral corner present, which tapers in width anteriorly and ventrally, and the angle between the dorsal and anterior face of the process rounded over (character 133), the posterior part of the suture between the nasals marked by a deep cleft (character 134), the postglenoid process ventrally tapers (character 169), the stylomastoid fossa situated on the posterior face of the pars cochlearis, posterodorsal to the stapedial muscle fossa (character 258), the dorsal edge of the tegmen tympani lateral to the aperture for the vestibular aqueduct presents as a faint ridge (character 273), and the deltopectoral tuberosity of humerus proximodistally centered (character 355).

Type genus

Delphinodon Leidy, 1869.

Included genera

Atocetus de Muizon, 1988, *Delphinodon* Leidy, 1869, *Hadrodelphis* Kellogg, 1966, *Lophocetus* Cope, 1868, *Liolithax* Kellogg, 1931, *Macrokentriodon* Dawson, 1996, *Tagicetus* Lambert et al., 2005, and *Platysvercus* gen. nov.

Etymology of family

The family name comes from the type genus, *Delphinodon*.

Genus *Platysvercus* gen. nov.

LSID. urn:lsid:zoobank.org:act:534A947D-EACC-406A-A853-D9D6041ADEE4

Type and only species

Platysvercus ugonis, sp. nov.

Diagnosis of genus

As for the type and only included species.

Etymology of genus

The genus *Platysvercus* is from Greek Platys, for broad or wide, and Greek Sverkos, for the back of the neck referring to the transversely wide nuchal crest of the new genus

Platysvercus ugonis sp. nov.

(Figs 2–6; Table 1)

LSID. urn:lsid:zoobank.org:act:141138C8-3D58-4852-80AD-3A5E8237CCD2

Fig 2. Dorsal view of the skull of *Platysvercus ugonis* gen. et sp. nov., holotype, UTHFM 00034. (A) photo. (B) corresponding line drawing with anatomical interpretations. Scale bar equals 100 mm.

Fig 3. Right lateral view of the skull of *Platysvercus ugonis* gen. et sp. nov., holotype, UTHFM 00034. (A) photo. (B) corresponding line drawing with anatomical interpretations. Scale bar equals 100 mm.

Fig 4. Ventral view of the skull of *Platysvercus ugonis* gen. et sp. nov., holotype, UTHFM 00034. (A) photo. (B) corresponding line drawing with anatomical interpretations. Scale bar equals 100 mm.

Fig 5. Posterior view of the skull of *Platysvercus ugonis* gen. et sp. nov., holotype, UTHFM 00034. (A) photo. (B) corresponding line drawing with anatomical interpretations. Scale bar equals 100 mm.

Fig 6. Posterior view from the cross section of rostrum *Platysvercus ugonis* gen. et sp. nov., holotype, UTHFM 00034, 50 mm anterior to the antorbital notch. (A) photo. (B) corresponding line drawing with anatomical interpretations. Scale bar equals 20 mm.

Table 1. Measurements (in mm) for the skull of *Platysvercus ugonis* gen et sp. nov., holotype, UTHFM 00034.

Dimension	Measurement
Condylobasal length, from tip of rostrum to hindmost margin of occipital condyles	566+
Length of rostrum, from tip to line across hindmost limits of antorbital notches	318.5+
Width of rostrum at base, along line across hindmost limits of antorbital notches	152e
Width of rostrum at 60 mm anterior to line across hindmost limits of antorbital notches	89.3
Width of rostrum at mid–length	54+
Width of premaxillaries at mid–length of rostrum	30.1
Width of rostrum at 3/4 length, measured from posterior end	49
Distance from tip of rostrum to external nares (to mesial end of anterior margin of right naris)	414+
Distance from tip of rostrum to internal nares (to mesial end of posterior margin of right pterygoid)	382+
Distance between first posterior alveolus and antorbital notch	58.9+
Distance between anterior tips of right and left premaxillary foramina	26.7
Projection of premaxillae beyond maxillae measured from tip of rostrum to line across foremost tips of maxillae visible in dorsal view	12.5+
Width of premaxillae at posterior extremity	49.6
Maximum width of mesorostral groove on the rostrum	12.5
Width of mesorostral groove at rostrum base	12.5
Width of right premaxillary sac fossa	37

Dimension	Measurement
Width of left premaxillary sac fossa	36.6
Length of medial suture of nasals	4.7
Width of nasal bones	43.3
Maximum length of frontal at the vertex	24.4
Greatest preorbital width	235.8e
Greatest postorbital width	261.8e
Least supraorbital width	224.2e
Greatest width of external nares	36.1
Greatest width across zygomatic processes of squamosals	237.6e
Greatest width of premaxillae	96
Greatest parietal width	181.6
Greatest length of temporal fossa, measured to external margin of temporal crest	144.3
Greatest width of temporal fossa perpendicular to greatest length	43.6
Major diameter of temporal fossa proper	52.1
Minor diameter of temporal fossa proper	82.9+
Distance from foremost end of junction between nasals to hindmost point of margin of nuchal crest	41.1
Length of orbit–from apex of preorbital process of frontal to apex of postorbital process	57
Length of antorbital process of lacrimal	31.7+
Greatest width of internal nares	54.2+
Width across occipital condyles	84.2
Width of the foramen magnum	32.3
Height of right occipital condyle	54.5
Width of right occipital condyle	31.8
Maximum distance between basioccipital crests	102.3+

Abbreviations: e, estimate; +, not complete

Holotype

UTHFM 00034, a nearly complete skull, discovered and collected by Toichiro Shibata in June 1992.

Diagnosis of Species

It differs from other species within the family in having a unique combination of ~~the following~~ characters: the premaxillary foramen located midpoint to lateral on the premaxilla (character 70), the premaxillae raised up towards the midline but concave transversely between the anteromedial sulci emanating from the premaxillary foramen

(character 121), and the anterior margin of the pterygoid sinus fossa located posterior to the antorbital notch (character 190).

Etymology of species

The species name *ugonis* is derived from Ugo Town, where the holotype was discovered and collected.

Type locality

The holotype was collected from a road construction site in Tashiro district, Ugo Town, Ogachi County, Akita Prefecture, Japan (39°21'57‘’N, 140°30'55‘’E; Fig 1).

Formation and age

Sugota Formation, early Miocene in age. The holotype was collected from the lowest part of the outcrop along the roadside during road construction. Judging from the stratigraphic horizon at the outcrop, this specimen was derived from the main part of the Sugota Formation. According to Sato et al. [35], Iwasa and Kikuchi [36], Fujioka et al. [37], and Aita [38], the main part of the Sugota Formation is correlated with the NN4–NN5 calcareous nannofossil zone and N9–N10 planktonic foraminiferal zone. Also, according to Aita [38], the Sugota Formation has produced *Praeorbulina sicana* and *Fohsella birnageae*, which is correlated with the M4b–M5a planktonic foraminiferal zone [39], ranging in age between 16.70 and 16.29 Ma, late Burdigalian, late early Miocene. The Sugota Formation is mainly composed of sand-stone with abundant mollusks [40]. Further, some *Desmostylus* teeth ~~have been~~ known from this Formation [41].

Description

Cranium

The cranium is nearly complete, and only the left antorbital notch, the right glenoid process of the squamosal, and most ~~parts~~ of the pterygoid are missing. Although the apex of the rostrum was slightly broken, it is very long. Deformation is observed at the dorsoventral level of the supraoccipital, temporal fossa, and occipital condyle. In the posterior view, the cranium is deformed; the higher part of the cranium is turned clockwise and skewed to the right, and the posteroventral part of the cranium is higher on the right side than the left. The nasals and the premaxillae are ~~structurally~~ symmetrical in the dorsal view, while the vertex is slightly skewed right to the midline of the occipital condyle because of this deformation. The temporal fossa is dorsoventrally narrow in ~~the~~ lateral view. The dorsal wall of the temporal fossa is low and flat, and it is almost horizontal at the level of the orbital fossa, and only shows slight~~ly~~ posterior elevation.

Premaxilla

The premaxillae are well preserved, and only the anterior tip ~~is~~ missing. The dorsal surface of the premaxillae is flat ~~at~~ the anterior rostral portion, while the flat area is rotated to face dorsolaterally, and its medial margin makes a sharp keel dorsally. The mesorostral groove opens along the medial margins of the premaxillae, starting from the level of the prenarial triangle, just anterior to the premaxillary foramen. In the ~~anteroposterior view~~ of the cross section of the rostral base (Fig 6), the mesorostral groove is U–shaped, and the medial edge of the premaxilla is slightly extended dorsomedially. The width of the premaxilla is narrow anteriorly and gradually ~~wider~~ posteriorly to the level of the prenarial triangle. Medially, the premaxillae do not cover the external bony nares and shapes following the anterolateral wall of the external bony nares. In ~~the~~ dorsal view, the external bony nares are formed anterolaterally by the premaxillae and posteriorly by the nasals and the presphenoids, and it is somewhat shaped like a heart–ma[illegible] The premaxillary foramen is positioned at the midpoint ~~of~~ the lateral and medial margins of the premaxilla and is located at the anterior margin of the premaxillary sac fossa and medial to the anteromedial sulcus. The premaxillary sac fossa is flat and gradually thickened posteriorly. Medially, it is higher than the portion lateral to the posterolateral sulcus of the premaxilla. The premaxilla contacts the nasal posteriorly, and the posterior end of the premaxilla is knob–like. The premaxillae are symmetrical at the vertex; however, at the level just anterior to the prenarial triangle, the right premaxilla is slightly wider than the left. The posterior margin of the premaxilla is distinctly higher than that of the maxilla in ~~the~~ lateral view.

Maxilla

The maxilla is well preserved, and only the anterior tip of the rostrum and the left antorbital process are missing. In ~~the~~ dorsal view, the maxilla is exposed ~~at~~ the rostral portion, and it is almost the same width as the premaxilla. The lateral margin of the maxilla is slightly concave laterally. The surface of the maxilla faces dorsally at the rostral base, whereas it ~~is located~~ dorsolaterally toward the anterior tip of the rostrum, as is also the case for the premaxilla. In the broken section at the rostral base (Fig 6), the ventral surface of the maxilla is convex, while the dorsal surface is flat or slightly concave. Three infraorbital canals are preserved in the broken section, one positioned in the suture between the premaxilla and the vomer, and two in the maxilla, where they are closely located to the maxilla–vomer suture.

The antorbital notch is triangular and posteriorly deep. Although the anterior edge is slightly damaged, the shape of the right antorbital process is somewhat rectangular. There are four anterior and one posterior dorsal infraorbital foramina; the anterior dorsal infraorbital foramina are located at the level of the prenarial triangle of the premaxilla, and the posterior dorsal infraorbital foramen is situated at the level of the external bony nares on the left maxilla while absent on the right one. The dorsal surface of the maxilla is flat ~~at the orbital area~~. The maxillary crest is poorly defined posterior to the antorbital process.

The facial fossa is shallow. The maxilla overlaps the frontal completely, and only ~~the~~ part of the postorbital process can be observed in ~~the~~ dorsal view. Posteriorly, the maxilla is somewhat square in ~~the~~ dorsal view. The maxilla contacts the supraoccipital posteriorly, and its posterolateral margin reaches the anteroposterior level of the jugular notch of the exoccipital. The nuchal crest is straight and forms a frontal–maxillary suture. At the vertex, the maxilla is lower than the posterior end of the premaxilla, nasal, frontal, and nuchal crests. It lies anterior to the nuchal crest and forms a deep fossa ~~at the portion~~ lateral to the frontal and posterior to the nasal and premaxilla. In ~~the~~ lateral view, the lateral margin of the maxilla from the level of the antorbital notch to the level of the ascending process is thin and flat.

In ~~the~~ ventral view, the left and right maxillae contact ~~to~~ each other at the midline, but they are not fused at the posterior half of the rostrum, while the maxilla is separated by the exposure of the palatine process of the premaxilla ~~at~~ the anterior half of the rostrum. Both sides of the maxilla preserve 28 tooth counts, and the total length of the maxillary alveolar groove is 270 mm. Each alveolus is small and almost the same size from the apex to the posterior part of the alveoli. The transverse diameters of each alveolus range from 4 mm to 6 mm. The maxillary–palatine suture cannot be defined. The anterior sinus fossa is shallow, located at the same level or slightly posterior to the antorbital notch. Posterior to the anterior sinus fossa, the ventral infraorbital foramen is positioned immediately lateral to the pterygoid fossa, while the surrounding bones are not clear because the pterygoid and lacrimal are damaged.

Palatine–Pterygoid

The maxillary–palatine suture is not preserved, and the anterior margin of the palatine is unclear. A fragment of the lateral lamina of the palatine is preserved and located immediately anterolateral to the external bony nares, but it is limited by the lateral width of the pterygoid sinus fossa. Most parts of the pterygoid are missing, and it only preserves its posterior lamina beside the vomer, although its suture with the vomer is unclear. The pterygoid sinus fossa is short. Its anterior wall is rounded and is extended posterior to the antorbital notch. The choana are narrower than the pterygoid sinus fossa.

Vomer

The external bony nares are still ~~stuffed by the~~ hard ~~nodule~~, so the vomer is not observable in ~~the~~ dorsal view. In the cross section of the rostral base, the dorsal surface of the vomer is U–shaped. It contacts the premaxilla dorsally and is surrounded by the maxilla ventrolaterally. A vomer–maxilla suture can be observed. In ~~the~~ ventral view, the vomer forms the medial wall of the choanae. Posteriorly, the vomer–basisphenoid suture is not clearly defined.

Presphenoi[illegible]

The nasal septum is low, and it contacts the presphenoid posteriorly. The nasal septum is dorsally lower than the dorsal surface of the surrounding premaxilla by 15 mm. Posteriorly, the presphenoid is high and is in contact with the nasal. In ~~the~~ dorsal view, the presphenoid is convex and triangular in shape. It forms a sharp keel anterodorsally (11 mm), while the transverse width (21 mm) is narrow.

Nasal

The nasal lies just behind the external bony nares and is surrounded laterally by the premaxilla and posteriorly by the frontal. In ~~the~~ dorsal view, the left and right nasals are symmetrical. The shape of each nasal is somewhat triangular in outline and pointed posteriorly towards its midpoint. Medially, the nasal ~~makes~~ contact with each other and forms a short suture. The nasal reaches ~~the~~ maximum width at the anterior margin, and it is slightly wider than the external bony nares. The dorsal surface of the nasal is smooth and only slightly concave anteromedially. Both the anterodorsal crest and the internasal fossa are not distinct. In ~~the~~ anterodorsal view, the internal surface of the nasal is narrow and short, and it only extends ventrally from the anterolateral edge.

Frontal

The frontal is almost fully covered ~~with~~ the maxilla and only exposed at the vertex and the lateral part of the supraorbital margin ~~in the dorsal view~~. It contacts with the nasal anteriorly and the nuchal crest of the supraoccipital posteriorly. The frontal is anteriorly pointed, posteriorly pinched by the maxilla, while it widens again and contacts the supraoccipital at the posterior–most margin. In ~~the~~ lateral view, the frontal at the vertex is lower than the nuchal crest and slightly lower than the nasal crest. The left and right frontal sutures can be observed in the dorsal view at the anterior part of the frontal, and it continues to the internasal suture.

The orbit is located along the lateral edge of the posterior rostrum. It is low and shallow dorsoventrally in the righ[illegible]ontal. The preorbital process is convex ventrally and covered with ~~a fragment of the~~ lacrimal. There is no ventral orbital crest. The postorbital process is poorly preserved on the right side, and it only remains as a blunt infratemporal crest at the base. It is located at ~~a~~ level just anterior to the choana. The optic canal ~~can~~ only be ~~clearly~~ distinct on the right side; it is located just lateral to the choana. The orbital fissure is located posteromedial to the postorbital process. ~~Departing~~ from the orbital fissure, the frontal groove is deep and wide (18 mm), and its anterior edge makes contact with the infratemporal crest. The foramen rotundum is positioned just anterior to the orbital fissure and is narrower than the latter.

Lacrimojugal Complex

The portion of the left antorbital process is damaged and only remains an unclear suture of the lacrimal, while the anterior edge of the right antorbital process of the lacrimal, which is also slightly damaged, is projected anterolaterally, and remains as a fragment beneath the process. It is thin in the supraorbital area and extends posteriorly to the anteroposterior level of the anterior wall of the choana. The jugal is not preserved on either side.

Squamosal

The right zygomatic process of the squamosal is almost broken, while it is well preserved on the left ~~one~~, only missing its anterior tip. In ~~the~~ dorsal view, the zygomatic process is slender, and its anterior end extends to the level of the choana in lateral view, just slightly posterior to the infratemporal crest. The supramastoid crest and lateral half of the glenoid process can be observed in ~~the~~ dorsal view. The supramastoid crest is parallel to the lateral margin of the maxilla and is almost parallel to the anteroposterior axis. The squamosal fossa is dorsoventrally deep (7 mm) and laterally wide (23 mm), and it faces dorsally. In the lateral view, the zygomatic process is rectangular and anteriorly slightly elevated dorsally.

In ~~the~~ ventral view, the mandibular fossa is large but shallow, and it faces medioventrally rather than anteroventrally. The post–tympanic process is shallow. It is laterally ~~margined~~ by the tympanosquamousal recess and anteriorly by the mandibular fossa. The posterior part of the postglenoid process is low. The tympanosquamosal recess is located immediately posteromedial to the mandibular fossa, and it is anteroposteriorly long and posteriorly wide. The falciform process is long and slender, and it makes the anterior wall of the cranial hiatus. In ~~the~~ ventral view, the periotic fossa is rounded. The foramen ovale is preserved on each side of the alisphenoid, anterior to the periotic fossa ~~by 5 mm~~, and it is 3 mm in diameter. The squamosal is fused with the alisphenoid, exoccipital, and parietal, ~~but the sutures are unclear~~.

Supraoccipital

Owing to the deformation ~~at~~ the occipitals, the dorsal part of the supraoccipital is compressed and skewed clockwise to the right side. The supraoccipital forms the nuchal crest and occupies the highest point of the cranium. Dorsally, the nuchal crest is thick and flat anteroposteriorly and laterally, and it is not extended anteriorly. In ~~the~~ lateral view, the nuchal crest is appreciably higher than the maxilla and falls slightly anteriorly. In ~~the~~ posterior view, the dorsal edge of the nuchal crest is circular and slightly rectangular; the shape is not related to the dorsal shape of the maxilla immediately anterior to it.

In the lateral view, the supraoccipital shield is flat and faces more posteriorly rather than dorsally. Therefore, the nuchal crest is short anteroposteriorly and not convex posteriorly in the dorsal view. Slightly lower than the nuchal crest, there is a deep depression at the center of the supraoccipital surface. The external occipital crest and suture between the supraoccipital and exoccipital are not preserved.

Exoccipital

The exoccipital is fused with the basioccipital and squamosal. In the dorsal view, the occipital condyle is slightly separated from the cranium and pushed out posteriorly. This may be a result of deformation, because the crack on the exoccipital can be observed dorsally, laterally, and ventrally. In the posterior view, the foramen magnum is compressed dorsoventrally, and the left and right occipital condyles are turned clockwise. The surface of the occipital condyle is smooth, while the dorsal margin is damaged, and the dorsal condyloid fossa is unclear. A short pedicle of the occipital condyle (approximately 15 mm in width) is developed, and the intercondyloid notch is U–shaped.

The jugular notch is deep in the ventral view, whereas the hypoglossal foramen is unclear. The paroccipital process is well developed. It extends more ventrally than the basioccipital crest and laterally reaches the lateral edge of the maxilla. The paroccipital concavity is shallow. The posterolateral sinus fossa is preserved but shallow.

Basioccipital

Both the left and right basioccipital crests are damaged at their posteroventral margins. In the ventral view, it diverges posteriorly. The transverse width at the posterior end of the basioccipital crest is slightly wider than the occipital condyle. The basioccipital crest contacts the posterior lamina of the pterygoid anteriorly. It covers most of the periotic fossa. The ventral surface of the basioccipital is flat and has no indication of the muscular tubercle.

Results

Phylogeny

We found 52 most parsimonious trees with 3385 steps. The strict consensus on these trees is shown in Fig 7. The 50% majority-rule consensus tree is also shown in Supporting Information S1. Our resultant trees show almost the same topology as was proposed by Guo and Kohno [8]; that is, all the ‘kentriodontid’ species that were identified by previous studies consisted of a monophyletic clade, and were placed as the sister group to the Delphinoidea (Fig 7). Species of ‘kentriodontids’ were split into two sub–clades; i.e., the sub–clade that contains *Whimal*, *Kampholophos*, *Sophianacetus*, *Rudicetus*, and *Kentriodon*, and the sub–clade that contains *Delphinodon*, *Tagicetus*, *Macrokentriodon*, *Hadrodelphis*,

Liolithax, *Atocetus*, and *Lophocetus*. UTHFM 00034 described here was recognized as an independent clade and was located as a sister taxon to *Macrokentriodon*, *Hadrodelphis*, and *Liolithax* in the latter sub–clade.

Fig 7. Phylogenetic relationships of *Platysvercus ugonis* gen. et sp. nov. Strict consensus tree resulting from 52 most parsimonious trees with tree constraint by the molecular trees of McGowen et al. [24–26], 3385 steps long, with the consistency index = 0.197 and the retention index = 0.568. Numbers above the nodes are Bremer support. Numbers below nodes indicate bootstrap values (1,000 replicated). The values less than 50% were omitted. The interspecific relationships of the Physeteroidea, the Ziphiidae, the Delphinidae, the Phocoenidae and the Monodontidae were omitted and these taxa were integrated into families/superfamilies.

Paleobiogeography

The results of the ancestral distributional area reconstructions are shown in Fig 8 and Supporting Information S1. The ancestor of the clade Delphinida (i.e., ‘Kentriodontidae’ along with Delphinoidea and Inioidea) was inferred to have emerged in the North Pacific Ocean (node 192, A, probability = 100%). The common ancestor of the monophyletic ‘kentriodontids’ was also most likely diversified with the sister clade, that is, the Delphinoidea, in the North Pacific Ocean (node 191, A, probability = 100%). However, the ancestral taxon of the Delphinoidea is unknown until the late middle Miocene in the North Pacific Ocean and is considered to have diversified just before the extinction of the ‘kentriodontids’ based on fossil records (node 190, A, probability = 100%). After the emergence of the ‘Kentriodontidae’ in the North Pacific, they spread out into the North Atlantic and became globally distributed in the Northern Hemisphere (node 153, AB, probability = 100%). Afterward, the ‘kentriodontids’ were split into two sub–clades as the ‘North Pacific’ clade and the ‘North Atlantic’ clade, respectively (node 152, A, probability = 100%; node 141, B, probability = 100%). In this regard, the biogeographical event of this split into two sub–clades is interpreted as vicariance, as will be discussed below. Similar vicariance events were also recognized just before or at the start of the middle Miocene Climatic Optimum (MMCO) at approximately 17 Ma (Fig 8). These were represented by the North Pacific UTHFM 00034 and the North Atlantic *Liolithax pappus* within the ‘North Atlantic’ sub–clade and also by the North Atlantic *Kentriodon schneideri* and the North Pacific *K. hobetsu* in the ‘North Pacific’ sub–clade. After the second vicariance event, species within both sub–clades were diversified in the Northern Hemisphere, and the North Pacific genus *Atocetus* within the ‘North Atlantic’ sub–clade also extended into the South Pacific Ocean (node 135, AC, probability = 100%).

Fig 8. Paleobiogeography and diversification of kentriodontoids. (A) Summary of the topology, ancestral range reconstructions and paleobiogeography of the kentriodontoids. The topology of the Kentriodontoidea is based on our phylogenetic analysis (Fig. 7). Stratigraphic ranges of each species are based on previous studies (see Supplemental Data 1 Table S1). Pie charts at each node show the ~~ancestral range from the result of~~ ancestral range reconstructions, numbers below in the pie chart indicate node number (Supplemental Data 1 Fig. S4 for complete results). (B) Number of species richness based on first occurrences of each kentriodontoid at each stage in the Miocene. Species of the Delphinodontidae are brown in color and those of the Kentriodontidae are dark green in color. Abbreviations: MMCO, the middle Miocene Climatic Optimum; EAIS, expansion of the East Antarctic Ice Sheet.

Discussion

Phylogeny and refined classification of ‘kentriodontids’

All the ‘kentriodontids’ known at least from crania are recognized as monophyletic, and 12 synapomorphies (i.e., characters 3, 7, 56, 62, 104, 140, 157, 225, 227, 278, 326, and 328) support this clade (see also the Diagnosis of Superfamily and Supporting Information S1). UTHFM 00034 described here is recognized ~~as an independent clade~~ by three autapomorphies (i.e., characters 70, 121, and 190) within the ‘kentriodontids’ (see also the Diagnosis of Genus and Supporting Information S1). Accordingly, we propose *Platysvercus ugonis* as a new genus and species on the basis of UTHFM 00034 as the holotype within this clade.

The monophyletic ‘Kentriodontidae’ is placed as a sister group to the Delphinoidea, as has already been proposed by some earlier studies [5,6,8]. However, the contents of the ‘kentriodontids’ differ among them. Our analysis supported the results of Guo and Kohno (2021), and the monophyletic ‘kentriodontids’ include~~d~~ all of the species previously recognized as ‘kentriodontids’ by 12 synapomorphies as was pointed out above. It is almost the same robustness as the superfamily Delphinoidea, which is supported by 18 synapomorphies. In this regard, the ‘kentriodontid’ clade could be recognized as a superfamilial rank as is the case for their sister taxon, the Delphinoidea. Hence, we propose the Superfamily Kentriodontoidea, to include all the ‘kentriodontids.

Our phylogenetic analysis also indicates that the new clade Kentriodontoidea is divided into two sub–clades. The one includes *Kampholophos serrulus*, *Kentriodon diusinus*, *Ke. hobetsu*, *Ke. hoepfneri*, *Ke. nakajimai*, *Ke. pernix*, *Ke. schneideri*, *Ke. sugawarai*, *Rudicetus squalodontoides*, *Sophianacetus commenticius*, and *Wimahl*

chinookensis. Five synapomorphies (i.e., characters 183, 245, 264, 266, and 277) support this sub–clade (see also Supporting Information S1). Some earlier studies [4,42] included all the taxa of 'kentriodontids' in the family 'Kentriodontidae,' but Peredo et al. [5] suggested that the Kentriodontidae *sensu stricto* only included four genera of 'kentriodontids,' which is approximately identical to our new definition of the Kentriodontidae. Our analysis also confirmed their result and followed the family name for our resultant sub–clade as the Kentriodontidae *sensu* Peredo et al. [5] for this group.

The other one includes *Atocetus iquensis*, *Atocetus nasalis*, *Delphinodon dividum*, *Hadrodelphis calvertense*, *Liolithax pappus*, *Lophocetus calvertensis*, *Lo. repenningi*, *Macrokentriodon morani*, *Platysvercus ugonis* gen. et sp. nov., and *Tagicetus joneti*. Eight synapomorphies (i.e., characters 37, 70, 133, 134, 169, 258, 273, and 355) support this sub–clade (see also the Diagnosis of Delphinodontidae and Supporting Information S1). Given that this clade has as many synapomorphies as the family Kentriodontidae as well as the Delphinidae (three), Monodontidae (thirteen), and Phocoenidae (seven), this clade can also be recognized as a family within the Kentriodontoidea. Here, we propose the new family Delphinodontidae for this sub–clade because *Delphinodon dividum* is the first recorded taxon in this group.

The type genera and species of both families (i.e. *Kentriodon pernix* for the Kentriodontidae and *Delphinodon dividum* for the Delphinodontidae) are represented by diagnostic specimens including the skull, tympanoperiotic, forelimbs, and vertebrae. At present, these two families consist of only about 10 taxa that are fewer in comparison with other odontocete families, but the numbers of synapomorphies at each node (five in the Kentriodontidae and eight in the Delphinodontidae) confirm each clade as robust as the Delphinidae, Monodontidae, and Phocoenidae within the Delphinoidea, as mentioned above. Again, the number of unique characters for each family is almost identical to that of other families within the Delphinida, but each kentriodontoid family cannot be instinctively defined by any autapomorphic characters. Future investigations to identify unique or autapomorphic characters will clarify the robustness of their relationships.

Paleobiogeography of kentriodontoids.

As has been suggested by some of the previous studies [4,43,44], kentriodontoids were recognized to have distributed worldwide during the middle Miocene (Fig 8 and Supporting Information). The results of our study indicate that the common ancestor of the Kentriodontoidea emerged in the North Pacific Ocean and immediately spread out in the Northern Hemisphere. The initial diversification of the Kentriodontoidea in the Northern Hemisphere is interpreted to be the range expansion of their distribution. At the time of the first split of the ancestral kentriodontoids into two subgroups, one of which emerged in the North Pacific Ocean and became the ancestor of the Kentriodontidae. By contrast, another subgroup seems to have emerged in the North Atlantic Ocean and later evolved into the

Delphinodontidae. Both events occurred, at the latest, before the middle Miocene as a vicariance event for the ancestral kentriodontoids (Fig 8A: nodes 152 and 141).

After the first emergence of the Kentriodontidae and Delphinodontidae, members of both families diverged sequentially and globally in the North Pacific and the North Atlantic through the late early and the late middle Miocene, respectively (Fig 8A, B: nodes 151, 147, 144, 137, 135, and 134). The Central American Seaway was opened intermittently during the early and middle Miocene [15,45], and it allowed repeated current connections as global ocean circulation [16,46]. This meant that marine vertebrates were able to migrate into and/or between the Pacific Ocean and the Atlantic Ocean without formidable barriers as a land bridge between North and South America [47,48]. In addition, the dispersal of the Kentriodontidae is thought to be somewhat earlier than that of the Delphinodontidae based on fossil records (Fig 8B and Supporting Information). The first occurrences (or the oldest known records) of each species within the Kentriodontidae during the Miocene are summarised as follows: one taxon appeared in the Aquitanian, six taxa in the Burdigalian, two taxa in the Langhian, and three taxa in the Serravallian. On the other hand, the first occurrences (or the oldest known records) of species within the Delphinodontidae are as follows: three taxa in the Burdigalian, three taxa in the Langhian, one taxon in the Serravallian, and four taxa in the Tortonian. Both families of the Kentriodontoidea declined and became extinct in the Tortonian (Fig 8A, B: nodes 144 and 138).

Several previous studies have suggested that the diversification and/or extinction events that occurred in the Miocene were the result of global ocean climate changes [18,49–51], based on carbon and oxygen isotopic changes [14], which were related to Miocene ice–sheet expansion events [17,52,53]. Other previous research [54] also suggested that the sea surface temperatures decreased by about 4°C –7 °C in the North Pacific Ocean and the North Atlantic Ocean from the middle Miocene Climatic Optimum (MMCO, 16.9–14.7 Ma) to the beginning of the expansion of the East Antarctic Ice Sheet (EAIS, 14–13 Ma). However, the interaction between the diversification and extinction of kentriodontoids and these climatic events has not yet been proven to be the cause of the extinction of marine mammals. Nevertheless, the vicissitudes of Kentriodontoidea were obviously influenced by these climate events. That is, nine taxa emerged within the MMCO, (16.9–14.7 Ma), and seven taxa were extinct around the period of or just after EAIS (Fig 8A). These climatic events may not only be the cause of the rise and fall of the kentriodontoids, but may also be related to the turnover event with the delphinoids around the Serravallian ([55], Plate 16a). However, it is still difficult to reveal the function and response between global climate change and these faunal turnover events.

The faunal turnover events for the Kentriodontoidea must also be considered in a different perspective, especially in the context of their extinction. The oldest known record of their sister taxon, the family Delphinoidea, goes back to 11.29–11.25 Ma [56], in the Tortonian of the middle Miocene. Previous studies [12,13] have suggested that the origin and early diversification of the Delphinoidea were recognized in the middle to late Miocene.

Although Delphinoidea have been considered to have the same ecological niches as environmental preferences [10] and feeding strategies [11] with most of the Kentriodontoidea, the absence of the Delphinoidea in the middle Miocene might have been the result of niche partitioning based on our phylogenetic and paleobiogeographic analysis. Similar to some research [4], the results suggest that the Kentriodontoidea might have declined in their niche by the diversification of the Delphinoidea in the late Miocene. This also indicates that the early diversification of the Delphinoidea may have occurred as a result of this turn over event.

Conclusions

An extinct delphinidan *Platysvercus ugonis* was described as a new genus and species belonging in the Kentriodontoidea, new rank, based on a nearly complete skull, UTHFM 00034, from the upper lower Miocene Sugota Formation, Akita Prefecture, northern Japan. *Platysvercus ugonis* gen. et sp. nov. shares the kentriodontoid synapomorphies, but it is distinguishable from other kentriodontoids by a unique combination of the following characters: the premaxillary foramen is located at the midpoint of the premaxilla; the premaxillae are raised up toward the midline and concave transversely, and the anterior level of the pterygoid sinus fossa is interrupted at the portion posterior to the antorbital notch. Based on our phylogenetic analysis, the systematics of the kentriodontoids was refined: i.e., the superfamily Kentriodontoidea mentioned above is proposed for all the species formerly included in the Kentriodontidae *sensu lato*. The new superfamily is subdivided into two families, the Kentriodontidae *sensu* Peredo et al. (2018) and the new family Delphinodontidae. *Platysvercus ugonis* gen. et sp. nov. is nested in the new family Delphinodontidae.

Our paleobiogeographic analysis of the new superfamily Kentriodontoidea indicates that their common ancestor emerged in the North Pacific Ocean and immediately spread out in the Northern Hemisphere. The initial diversification of the ancestral taxon or taxa of kentriodontoids is interpreted to be a result of the range expansion of their distribution in the Northern Hemisphere, and the Kentriodontids *sensu stricto* might have emerged and diversified in the North Pacific Ocean, while the Delphinodontidae fam. nov. might have emerged and diversified in the North Atlantic Ocean as a vicariance event for kentriodontoids. However, the Kentriodontoids decreased and became extinct following the climatic change during the middle Miocene, synchronously influenced by the expansion of the East Antarctic Ice Sheet. The Kentriodontoidea also seem to have declined in their niche due to the rapid diversification of the Delphinoidea during the middle to late Miocene boundary age as a turnover event around the Serravallian.

Acknowledgments

We wish to thank T. Shibata for discovering the holotype skull of *Platysvercus ugonis* gen. et sp. nov. described here for the UTHFM. We also thank K. Ito, F. Kakizaki, and S. Goto (both UTHFM) for permitting us to describe UTHFM 00034. We would like to thank Y. Tajima (NMNS) and T. Kimura (GMNH) for providing access to the collections under their care. We also thank Y. Tajima, H. Ichishima (Fukui Prefectural Dinosaur Museum), Y. Tanaka (Osaka Museum of Natural History), and T.K. Yamada (NMNS) for providing useful advice. We are grateful to K. Sashida (University of Tsukuba, now Mahidol University), S. Agematsu (Univ. Tsukuba) and K. Tanaka (Univ. Tsukuba), and I. Tanaka (Univ. Tsukuba, now the Geological Survey of Japan, AIST) and Y. Shigeta (NMNS/Univ. Tsukuba) for providing useful advice, discussion, and generous encouragement during the course of this study. Finally, the manuscript was greatly improved by careful attention to detailed and extensive comments from xxxx, xxxx, xxxx, and xxxx.

References

1. Barnes LG, Mitchell ED. *Kentriodon obscurus* (Kellogg, 1931), a fossil dolphin (Mammalia: Kentriodontidae) from the Miocene Sharktooth Hill Bonebed in California. Contrib. Sci. Nat. Hist. Mus. Los Angeles County. 1984;353: 1–23.

2. Barnes LG. The late Miocene dolphin *Pithanodelphis* Abel, 1905 (Cetacea, Kentriodontidae) from California. Contrib. Sci. Nat. Hist. Mus. Los Angeles County. 1985;367: 1–27.

3. Muizon C de. Les relations phylogénétiques des Delphinida (Cetacea, Mammalia). Ann. Paléontologie. 1988;74: 159–227.

4. Ichishima H, Barnes LG, Fordyce RE, Kimura M, Bohaska DJ. A review of kentriodontine dolphins (Cetacea; Deiphinoidea; Kentriodontidae): Systematics and biogeography. Isl. Arc. 1995;3: 486–492 (for 1994). doi:10.1111/j.1440-1738.1994.tb00127.x.

5. Peredo CM, Uhen MD, Nelson MD. A new kentriodontid (Cetacea: Odontoceti) from the early Miocene Astoria Formation and a revision of the stem delphinidan family Kentriodontidae. J. Vertebr. Paleontol. 2018;38. doi:10.1080/02724634.2017.1411357.

6. Lambert O, Bianucci G, Urbina M, Geisler JH. A new inioid (Cetacea, Odontoceti, Delphinida) from the Miocene of Peru and the origin of modern dolphin and porpoise families. Zool. J. Linn. Soc. 2017;179: 919–946. doi:10.1111/zoj.12479.

7. Kimura T, Hasegawa Y. A new species of *Kentriodon* (Cetacea, Odontoceti, Kentriodontidae) from the Miocene of Japan. J. Vertebr. Paleontol. 2019;39: 1–14. doi:10.1080/02724634.2019.1566739.

8. Guo Z, Kohno N. A new kentriodontid (Cetacea: Odontoceti) from the early to middle Miocene of the western North Pacific and a revision of kentriodontid phylogeny. PeerJ. 2021;9: e10945. doi:10.7717/peerj.10945.

9. Lambert O, Auclair C, Cauxeiro C, Lopez M, Adnet S. A close relative of the Amazon river dolphin in marine deposits: A new Iniidae from the late Miocene of Angola. PeerJ. 2018;6: e5556. doi:10.7717/peerj.5556.

10. Gutstein CS, Figueroa-Bravo CP, Pyenson ND, Yury-Yañez RE, Cozzuol MA, Canals M. High frequency echolocation, ear morphology, and the marine-freshwater transition: A comparative study of extant and extinct toothed whales. Palaeogeogr. Palaeoclimatol. Palaeoecol. 2014;400: 62–74. doi:10.1016/j.palaeo.2014.01.026.

11. Berta A, Lanzetti A. Feeding in marine mammals: An integration of evolution and ecology through time. Palaeontol. Electron. 2020;23: 1–42. doi:10.26879/951.

12. Murakami M, Shimada C, Hikida Y, Hirano H. Two new extinct basal phocoenids (Cetacea, Odontoceti, Delphinoidea), from the upper Miocene Koetoi Formation of Japan and their phylogenetic significance. J. Vertebr. Paleontol. 2012;32: 1172–1185. doi:10.1080/02724634.2012.694337.

13. Murakami M, Shimada C, Hikida Y, Soeda Y, Hirano H. Asymmetrical basal delphinoid skull from the upper lower Miocene Yamato Formation of Hokkaido, northern Japan: implications on evolution of cranial asymmetry and symmetry in Odontoceti. Paleontological Research. 2014;18: 134–149. doi:10.2517/2014pr013.

14. Kochhann KGD, Holbourn A, Kuhnt W, Channell JET, Lyle M, Shackford JK, Wilkens RH, Andersen N. Eccentricity pacing of eastern equatorial Pacific carbonate dissolution cycles during the Miocene Climatic Optimum. Paleoceanography. 2016;31: 1176–1192. doi:10.1002/2016pa002988.

15. Herold N, Seton M, Müller RD, You Y, Huber M. Middle Miocene tectonic boundary conditions for use in climate models. Geochemistry, Geophys. Geosystems. 2008;9. doi:10.1029/2008GC002046.

16. von der Heydt A, Dijkstra HA. Effect of ocean gateways on the global ocean circulation in the late Oligocene and early Miocene. Paleoceanography. 2006;21. doi:10.1029/2005PA001149.

17. Gasson E, DeConto RM, Pollard D, Levy RH. Dynamic Antarctic ice sheet during the early to mid-Miocene. Proc. Natl. Acad. Sci. U. S. A. 2016;113: 3459–3464. doi:10.1073/pnas.1516130113.

18. Böhme M. The Miocene Climatic Optimum: Evidence from ectothermic vertebrates of Central Europe. Palaeogeogr. Palaeoclimatol. Palaeoecol. 2003;195: 389–401. doi:10.1016/S0031-0182(03)00367-5.

19. Ichishima H. A new fossil kentriodontid dolphin (Cetacea; Kentriodontidae) from the middle Miocene Takinoue Formation, Hokkaido, Japan. Isl. Arc. 1995;3: 473–485 (for 1994). doi:10.1111/j.1440-1738.1994.tb00126.x.

20. Tanaka Y, Abella J, Aguirre-Fernández G, Gregori M, Fordyce RE. A new tropical Oligocene dolphin from Montañita/Olón, Santa Elena, Ecuador. PLoS One. 2017;12: e0188380. doi:10.1371/journal.pone.0188380.

21. Tanaka Y, Fordyce RE. Fossil dolphin *Otekaikea marplesi* (latest Oligocene, New Zealand) expands the morphological and taxonomic diversity of Oligocene cetaceans. PLoS One. 2014;9: e107972. doi:10.1371/journal.pone.0107972.

22. Geisler JH, Godfrey SJ, Lambert O. A new genus and species of late Miocene inioid (Cetacea, Odontoceti) from the Meherrin River, North Carolina, U.S.A. J. Vertebr. Paleontol. 2012;32: 198–211. doi:10.1080/02724634.2012.629016.

23. Geisler JH, McGowen MR, Yang G, Gatesy J. A supermatrix analysis of genomic, morphological, and paleontological data from crown Cetacea. BMC Evol. Biol. 2011;11. doi:10.1186/1471-2148-11-112.

24. McGowen MR, Spaulding M, Gatesy J. Divergence date estimation and a comprehensive molecular tree of extant cetaceans. Mol. Phylogenet. Evol. 2009;53: 891–906. doi:10.1016/j.ympev.2009.08.018.

25. Mcgowen MR, Montgomery SH, Clark C, Gatesy J. Phylogeny and adaptive evolution of the brain-development gene microcephalin (MCPH1) in cetaceans. BMC Evol Biol. 2011;11: 98 doi:10.1186/1471-2148-11-98.

26. McGowen MR et al. Phylogenomic resolution of the cetacean tree of life using target sequence capture. Syst. Biol. 2020;69: 479–501. doi:10.1093/sysbio/syz068.

27. Goloboff PA, Catalano SA. TNT version 1.5, including a full implementation of phylogenetic morphometrics. Cladistics. 2016;32: 221–238. doi:10.1111/cla.12160.

28. Goloboff PA, Farris JS, Nixon KC. TNT, a free program for phylogenetic analysis. Cladistics. 2008;24: 774–786. doi:10.1111/j.1096-0031.2008.00217.x.

29. Yu Y, Harris AJ, He X. S-DIVA (Statistical Dispersal-Vicariance Analysis): A tool for inferring biogeographic histories. Mol. Phylogenet. Evol. 2010;56: 848–850. doi:10.1016/j.ympev.2010.04.011.

30. Yu Y, Harris AJ, Blair C, He X. RASP (Reconstruct Ancestral State in Phylogenies): A tool for historical biogeography. Mol. Phylogenet. Evol. 2015;87: 46–49. doi:10.1016/j.ympev.2015.03.008.

31. Yu Y, Blair C, He X. RASP 4: Ancestral state reconstruction tool for multiple genes and characters. Mol. Biol. Evol. 2020;37: 604–606. doi:10.1093/molbev/msz257.

32. The IUCN red list of threatened species. Available from: https://www.iucnredlist.org/ (accessed on 31 January 2021.

33. Mead JG, Fordyce RE. The therian skull : a lexicon with emphasis on the odontocetes. Smithson. Contrib. to Zool. 2009;627: 1-248. doi:10.5479/si.00810282.627.

34. Ichishima H. 2016 The ethmoid and presphenoid of cetaceans. J. Morphol. 2016; 277: 1661–1674. doi:10.1002/jmor.20615.

35. Sato T, Baba K, Ohguchi T, Takayama T. Discovery of early Miocene calcareous nannofossils from Japan Sea side, northern Honshu, Japan, with reference to paleoenvironment in the Daijima and Nishikurosawa Ages. J. Japanese Assoc. Pet. Technol. 1991;56: 263–279. doi:10.3720/japt.56.263.

36. Iwasa S, Kikuchi Y. Foraminifera from the Sugota Formation, Akita Prefecture, Japan. Trans. Proc. Palaeont. Soc. Japan. 1954;16: 183–194. doi:10.14825/prpsj1951.1954.16_183.

37. Fujioka N, Ohguchi T, Maiya S, Usuda M, Baba K. On the sediments of Daijima-Nishikurosawan Age in the northeast inner Japan area. J. Japanese Assoc. Pet. Technol. 1981;46: 159–174. doi:10.3720/japt.46.159.

38. Aita Y. The lower Neogene of Dewa hilly land, Akita Prefecture, Japan. Annual Meeting of the Geological Society of Japan, 1984, The 91st Annual Meeting. 1984;121. [Japanese]

39. Wade BS, Pearson PN, Berggren WA, Pälike H. Review and revision of Cenozoic tropical planktonic foraminiferal biostratigraphy and calibration to the geomagnetic polarity and astronomical time scale. Earth-Science Rev. 2011;104: 111–142. doi:10.1016/j.earscirev.2010.09.003.

40. Ozawa A, Ohguchi T, Takayasu T. Geology of the Asamai district. Quadrangle Series, 1:50,000: Geol. Surv. Japan; 1979. [Japanese with English abstract]

41. Tan K, Shikama T. On *Desmostylus* teeth from Tashiro, Akita Prefecture. Sci. Rep. Yokohama Natl. Univ. 1965;2: 49–55. [Japanese with English abstract]

42. Barnes LG. A review of *Lophocetus* and *Liolithax* and their relationships to the delphinoid family Kentriodontidae (Cetacea: Odontoceti). Bull. Nat. Hist. Mus. Los Angeles Cty. 1978;28: 1–35.

43. Kazár E. A new kentriodontid (Cetacea: Delphinoidea) from the middle Miocene of Hungary. Foss. Rec. 2005;8: 53–73. doi:10.1002/mmng.200410004.

44. Kazár E, Hampe O. A new species of *Kentriodon* (Mammalia, Odontoceti, Delphinoidea) from the middle/late Miocene of Groß Pampau (Schleswig-Holstein, North Germany). J. Vertebr. Paleontol. 2014;34: 1216–1230. doi:10.1080/02724634.2014.857347.

45. Kirby MX, Jones DS, MacFadden BJ. Lower Miocene stratigraphy along the Panama Canal and its bearing on the Central American Peninsula. PLoS One. 2008;3: e2791. doi:10.1371/journal.pone.0002791.

46. Herold N, Huber M, Müller RD, Seton M. Modeling the Miocene climatic optimum: Ocean circulation. Paleoceanography. 2012;27. doi:10.1029/2010PA002041.

47. Lessios HA. The great American schism: Divergence of marine organisms after the rise of the Central American Isthmus. Ann. Rev. Ecol. Evol. Syst. 2008;39: 63–91. doi:10.1146/annurev.ecolsys.38.091206.095815.

48. Toonen RJ, Bowen BW, Iacchei M, Briggs JC. Biogeography, Marine. In: Encyclopedia of Evolutionary Biology: Elsevier; 2016. pp. 166–178 doi:10.1016/B978-0-12-800049-6.00120-7.

49. Marx FG, Uhen MD. Climate, Critters, and Cetaceans: Cenozoic Drivers of the Evolution of Modern Whales. Science. 2010;327: 993–996. doi:10.1126/science.1185581.

50. Johnson K, Hayward BW, Holbourn A. Impact of the middle Miocene climate transition on elongate, cylindrical foraminifera in the subtropical Pacific. Mar. Micropaleontol. 2011;78: 50–64. doi:10.1016/j.marmicro.2010.09.008.

51. Suto I, Kawamura K, Hagimoto S, Teraishi A, Tanaka Y. Changes in upwelling mechanisms drove the evolution of marine organisms. Palaeogeography, Palaeoclimatology, Palaeoecology. 2012;339–341: 39–51. doi:10.1016/j.palaeo.2012.04.014.

52. Flower BP, Kennett JP. Middle Miocene deep water paleoceanography in the southwest Pacific: Relations with East Antarctic Ice Sheet development. Paleoceanography 1995;10: 1095–1112. doi:10.1029/95PA02022.

53. Holbourn A, Kuhnt W, Schulz M, Erlenkeuser H. Impacts of orbital forcing and atmospheric carbon dioxide on Miocene ice-sheet expansion. Nature. 2005;438: 483–487. doi:10.1038/nature04123.

54. Frigola A, Prange M, Schulz M. Boundary conditions for the middle Miocene Climate Transition (MMCT v1.0). Geosci. Model Dev. 2018;11: 1607–1626. doi:10.5194/gmd-11-1607-2018.

55. Marx FG, Lambert O, Uhen MD. Cetacean Paleobiology: Wiley Blackwell, Topics in Paleobiology; 2016. doi:10.1002/9781118561546.

56. Kimura T, Hasegawa Y. *Norisdelphis annakaensis*, A new Miocene delphinid from Japan. J. Vertebr. Paleontol. 2020; e1762628. doi:10.1080/02724634.2020.1762628.

Supporting information

S1 File. Phylogenetic trees, list of taxa and characters states and materials and results of the ancestral range reconstructions.

S2 File. Data matrix used for the phylogenetic analysis in ".nex" format.

S3 File. Data matrix used for the phylogenetic analysis in ".tnt" format.

S4 File. README file including all the steps of the phylogenetic analysis.

S5 File. Tree file from phylogenetic analysis.

Figure 1
Click here to access/download;Figure;Fig1.tif

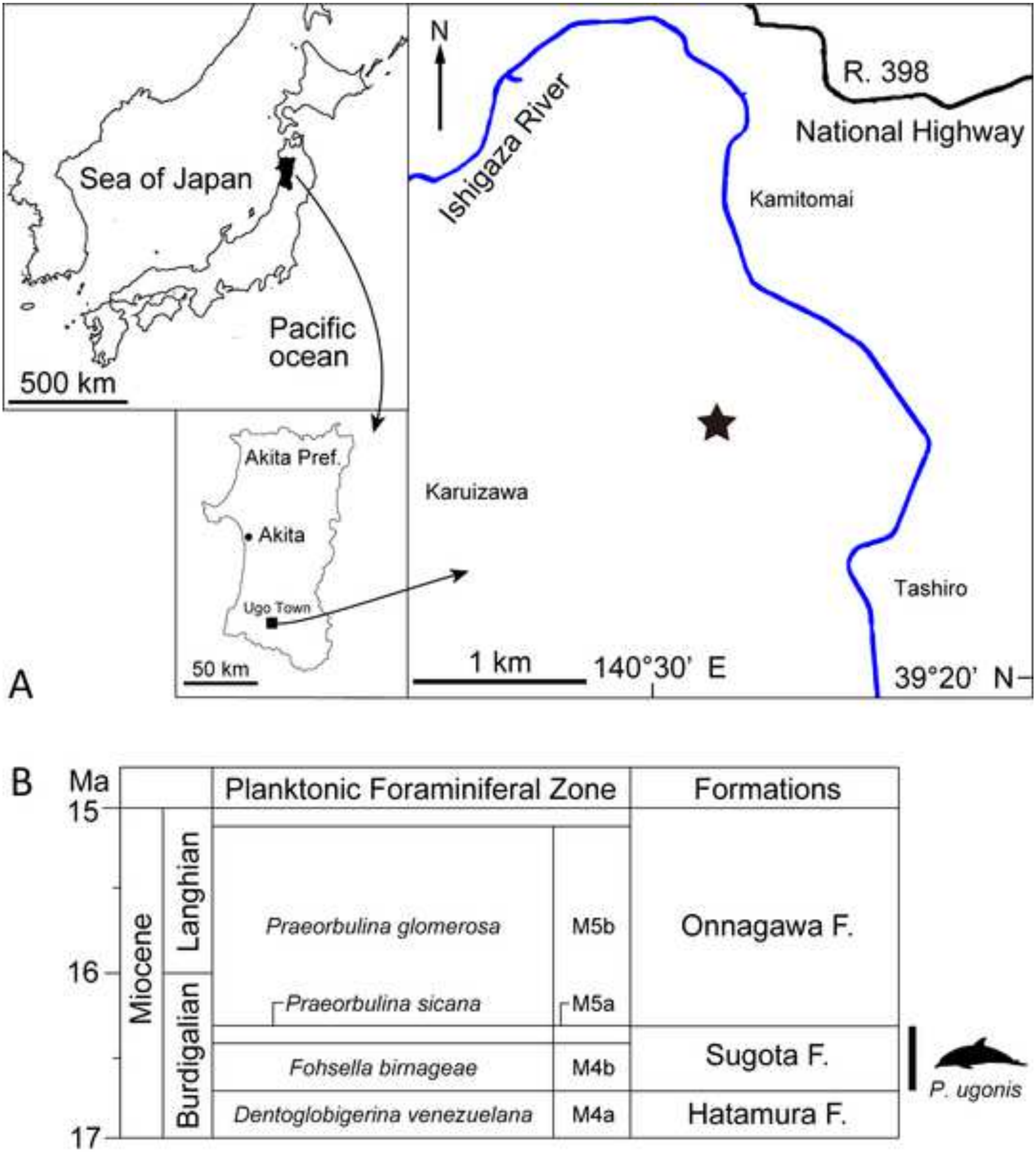

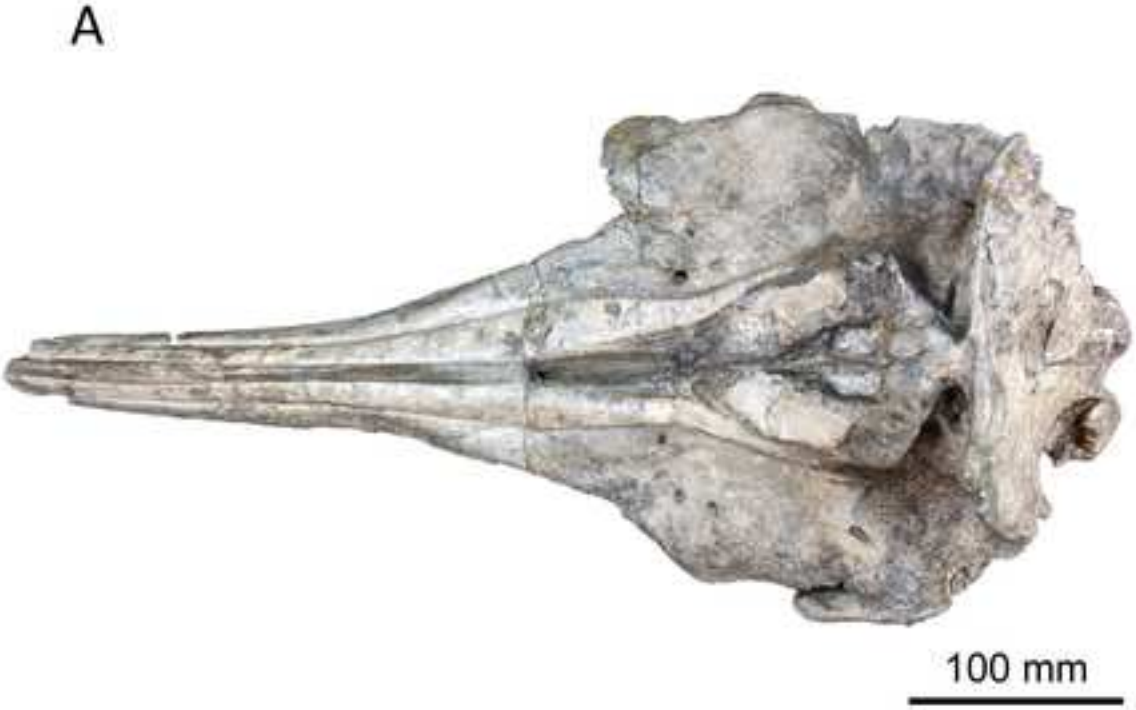
A
100 mm

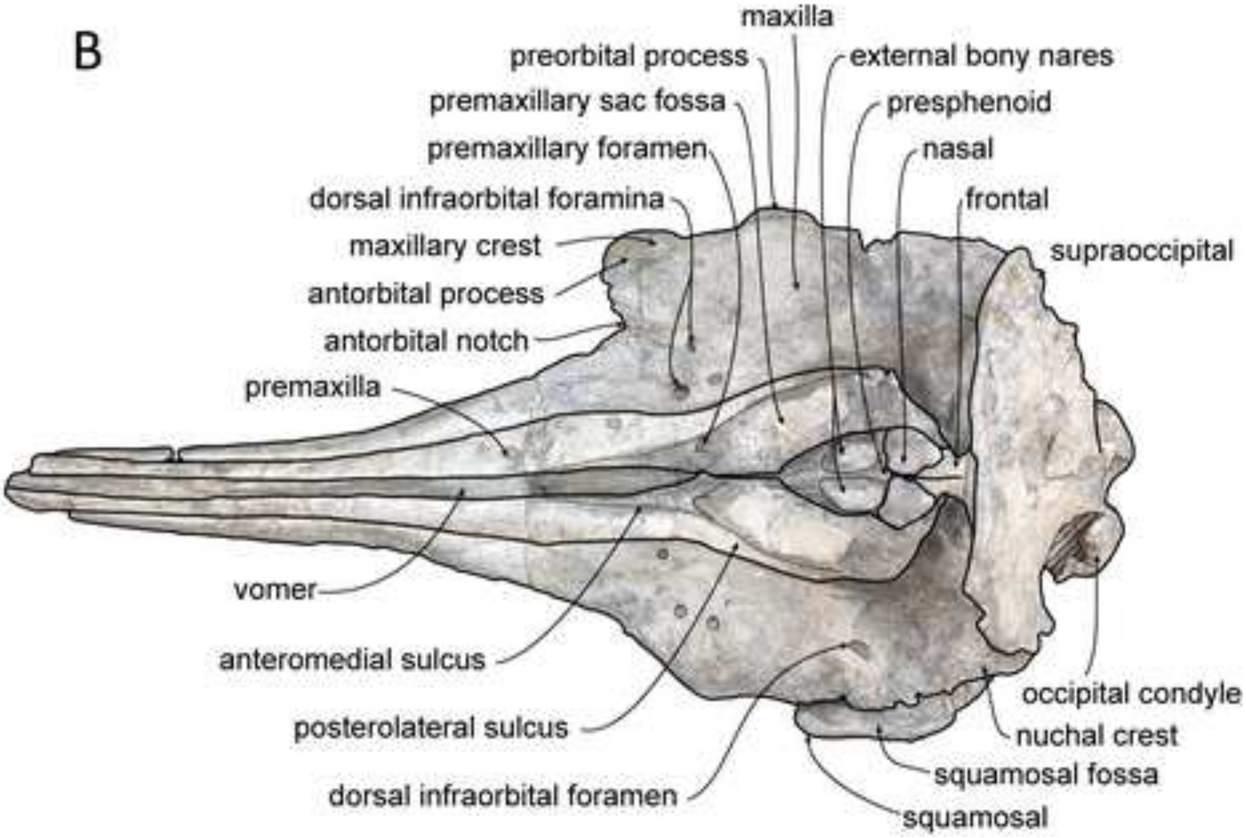
B
maxilla
preorbital process
external bony nares
premaxillary sac fossa
presphenoid
premaxillary foramen
nasal
dorsal infraorbital foramina
frontal
maxillary crest
supraoccipital
antorbital process
antorbital notch
premaxilla
vomer
anteromedial sulcus
posterolateral sulcus
dorsal infraorbital foramen
occipital condyle
nuchal crest
squamosal fossa
squamosal

Figure 3

Click here to access/download;Figure;Fig3.tif

A

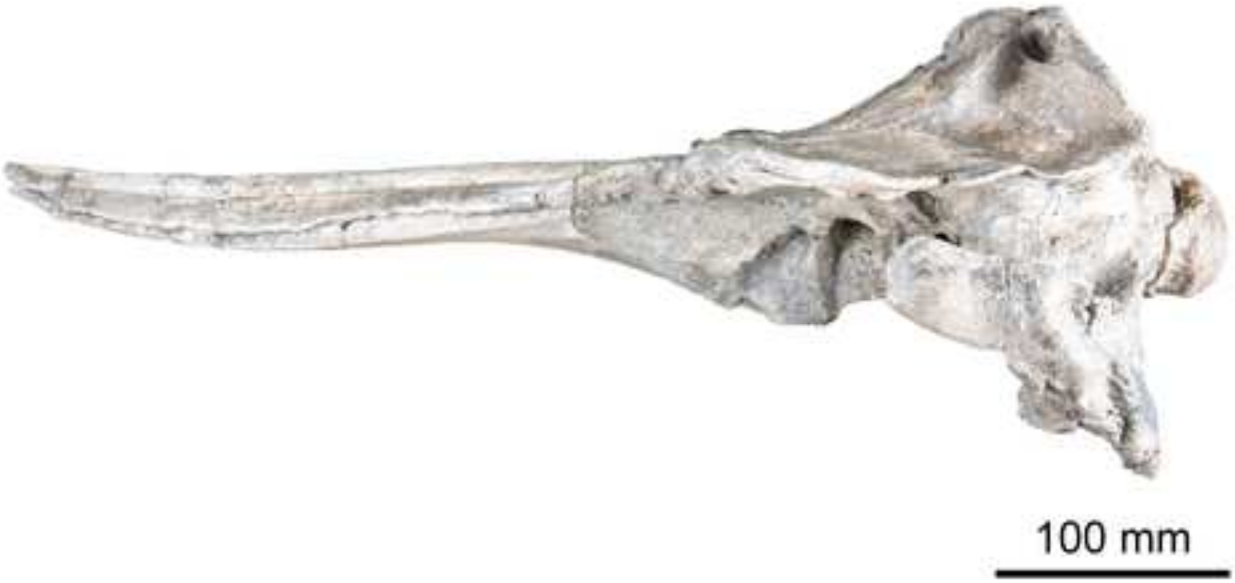

B

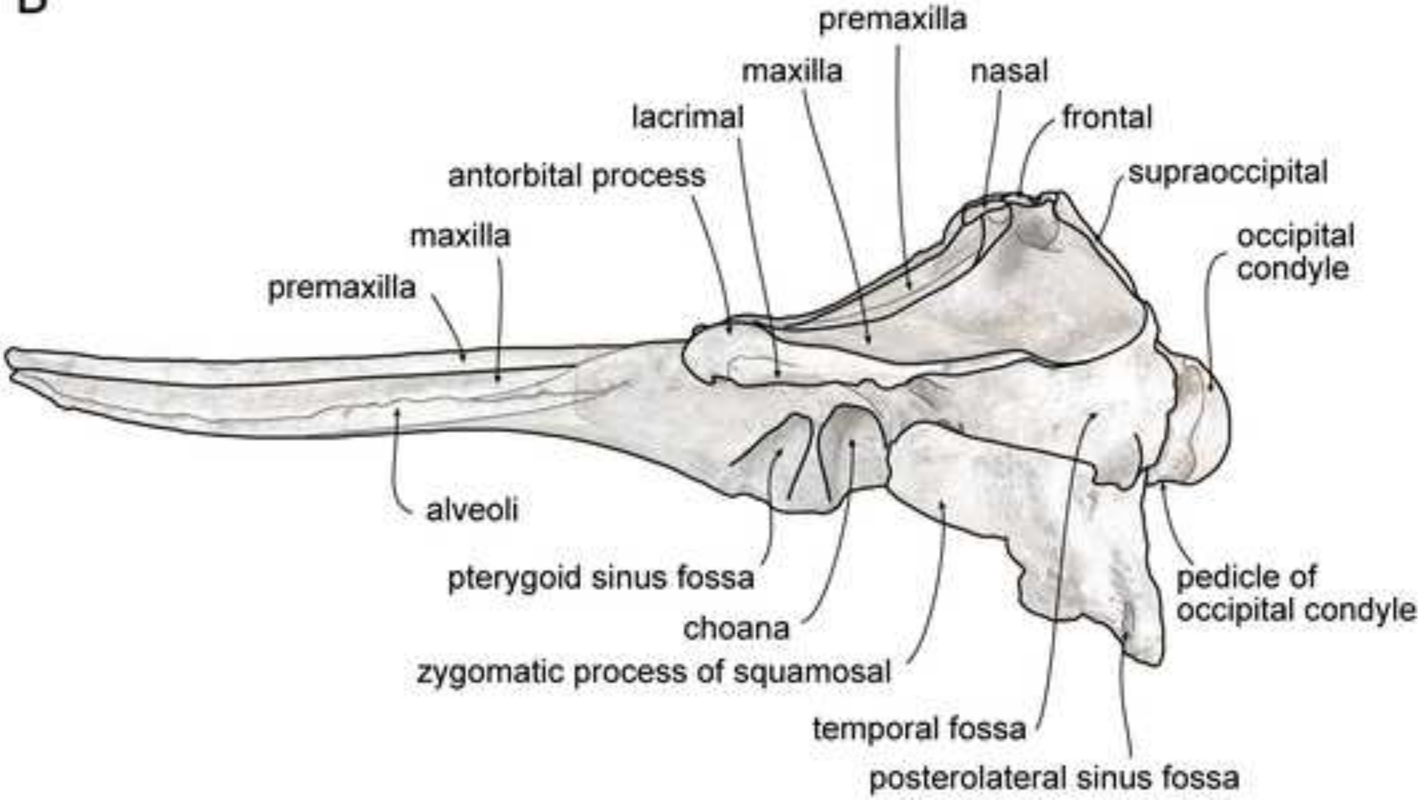

Figure 4

Click here to access/download;Figure;Fig4.tif

A

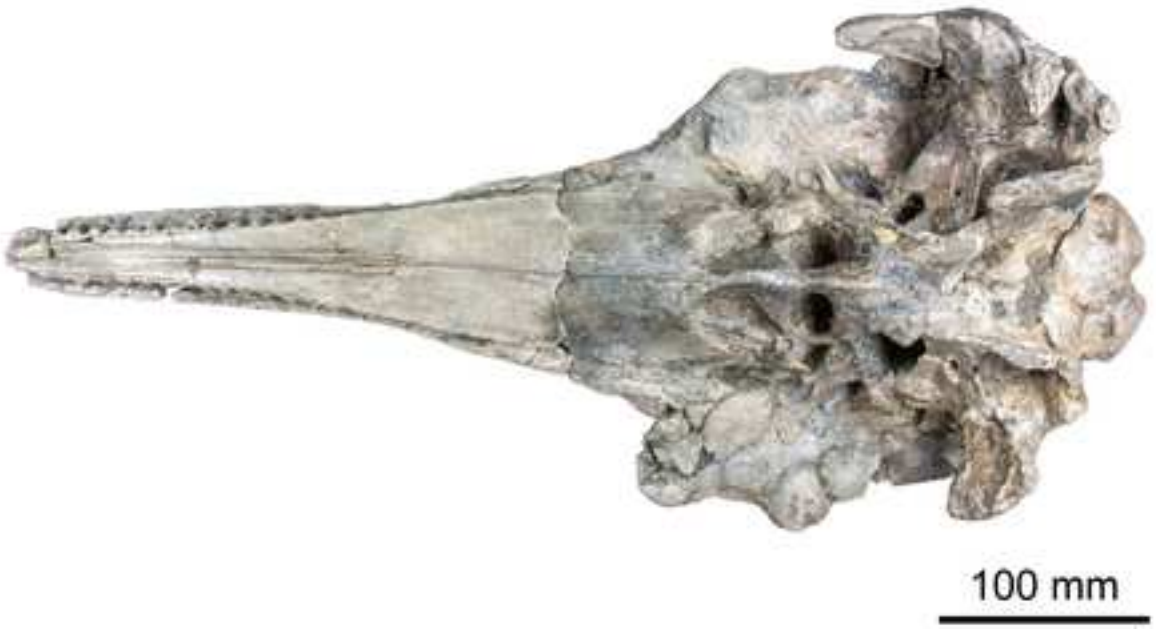

B

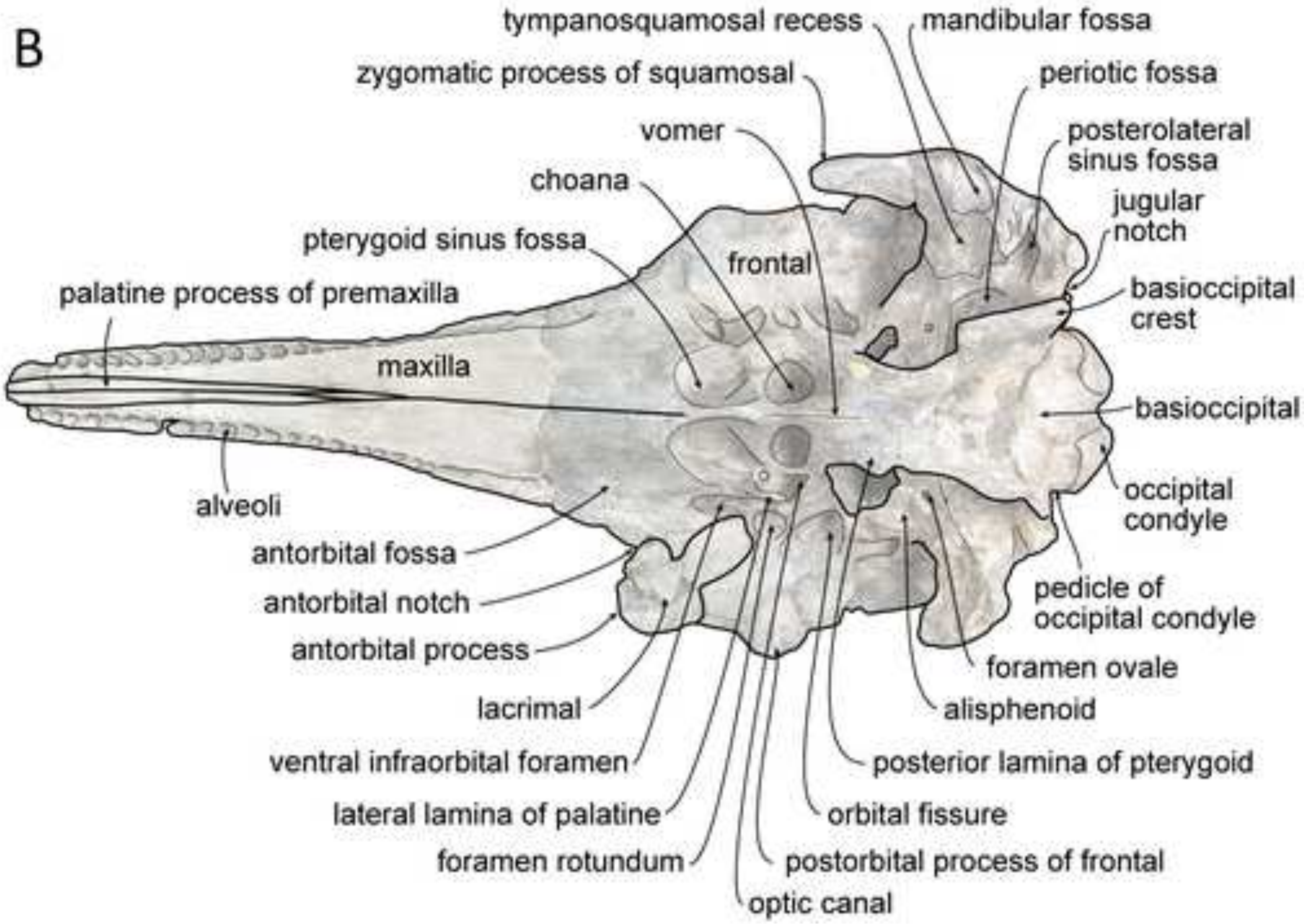

Figure 5

Click here to access/download;Figure;Fig5.tif

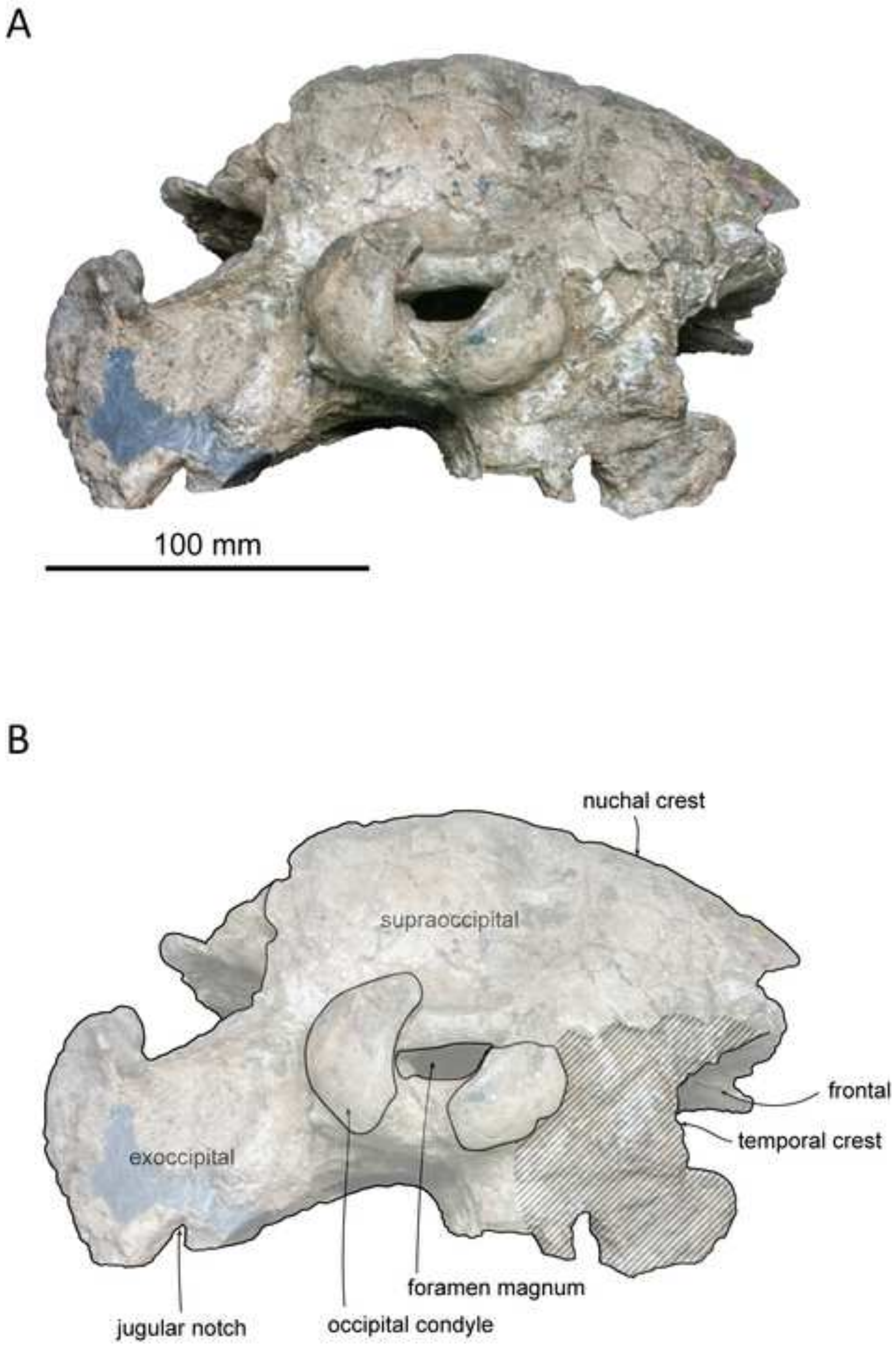

Figure 6

Click here to access/download;Figure;Fig6.tif

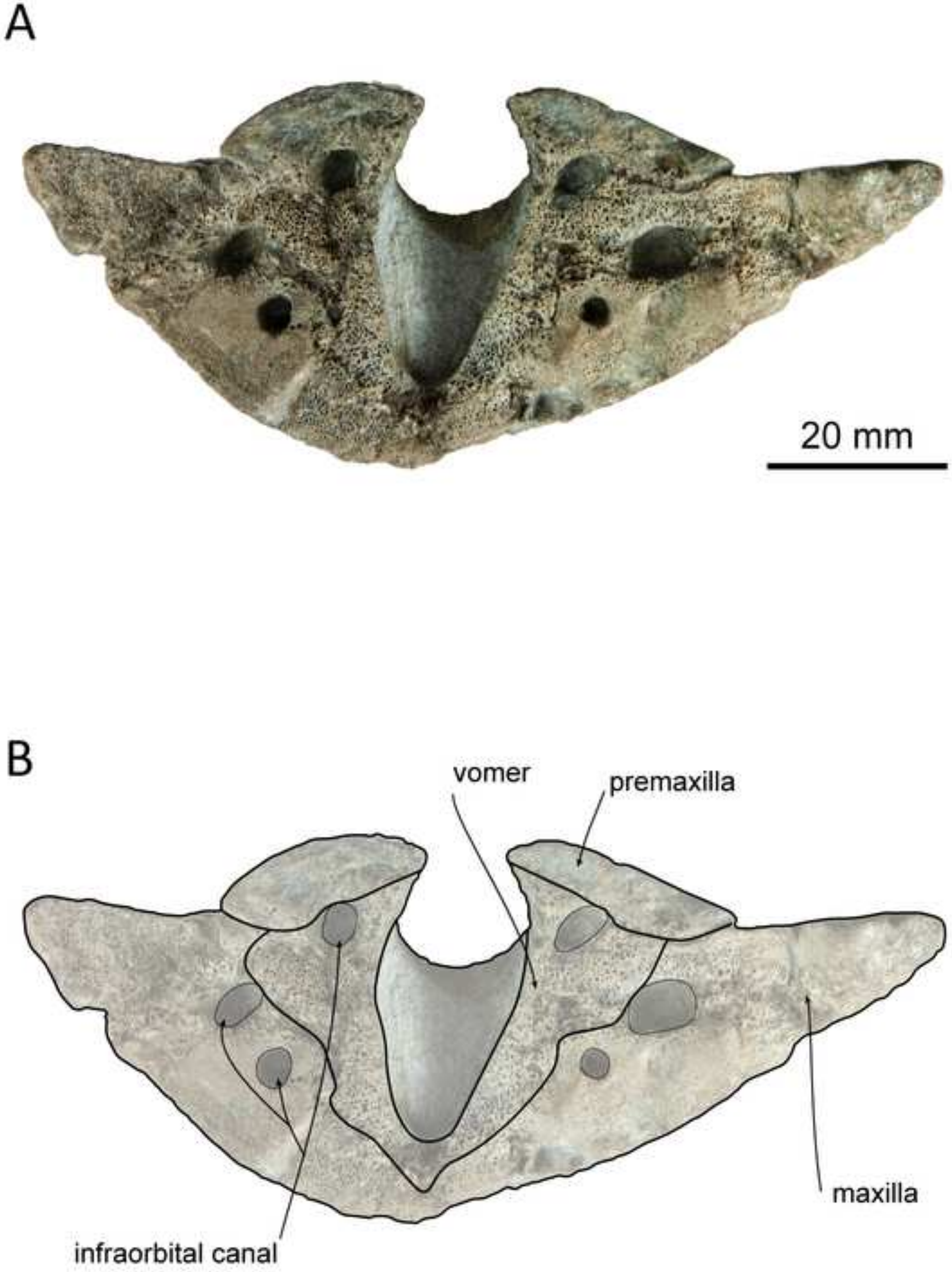

Figure 7 Click here to access/download;Figure;Fig7.tif

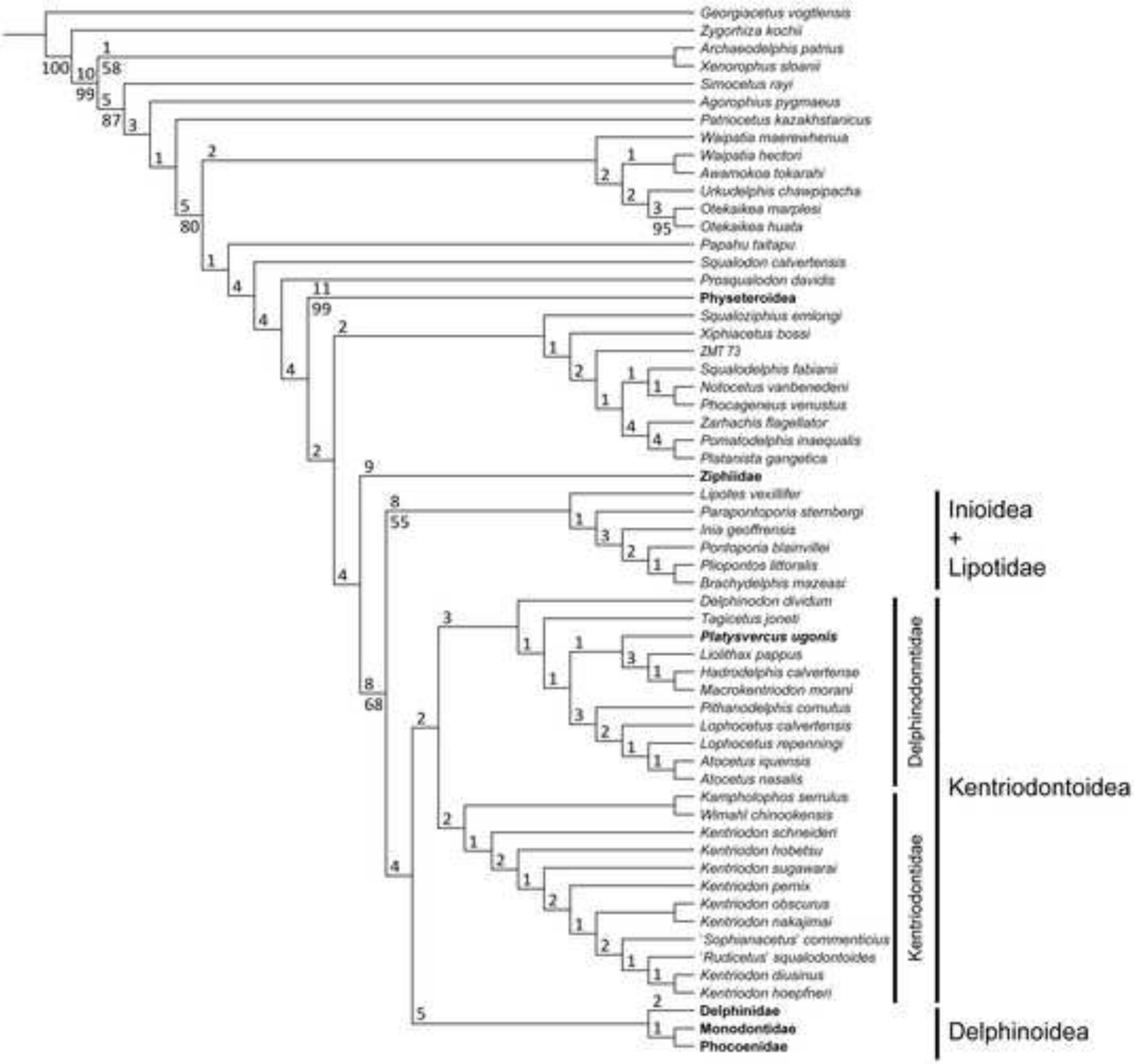

Figure 8

Click here to access/download;Figure;Fig8.tif

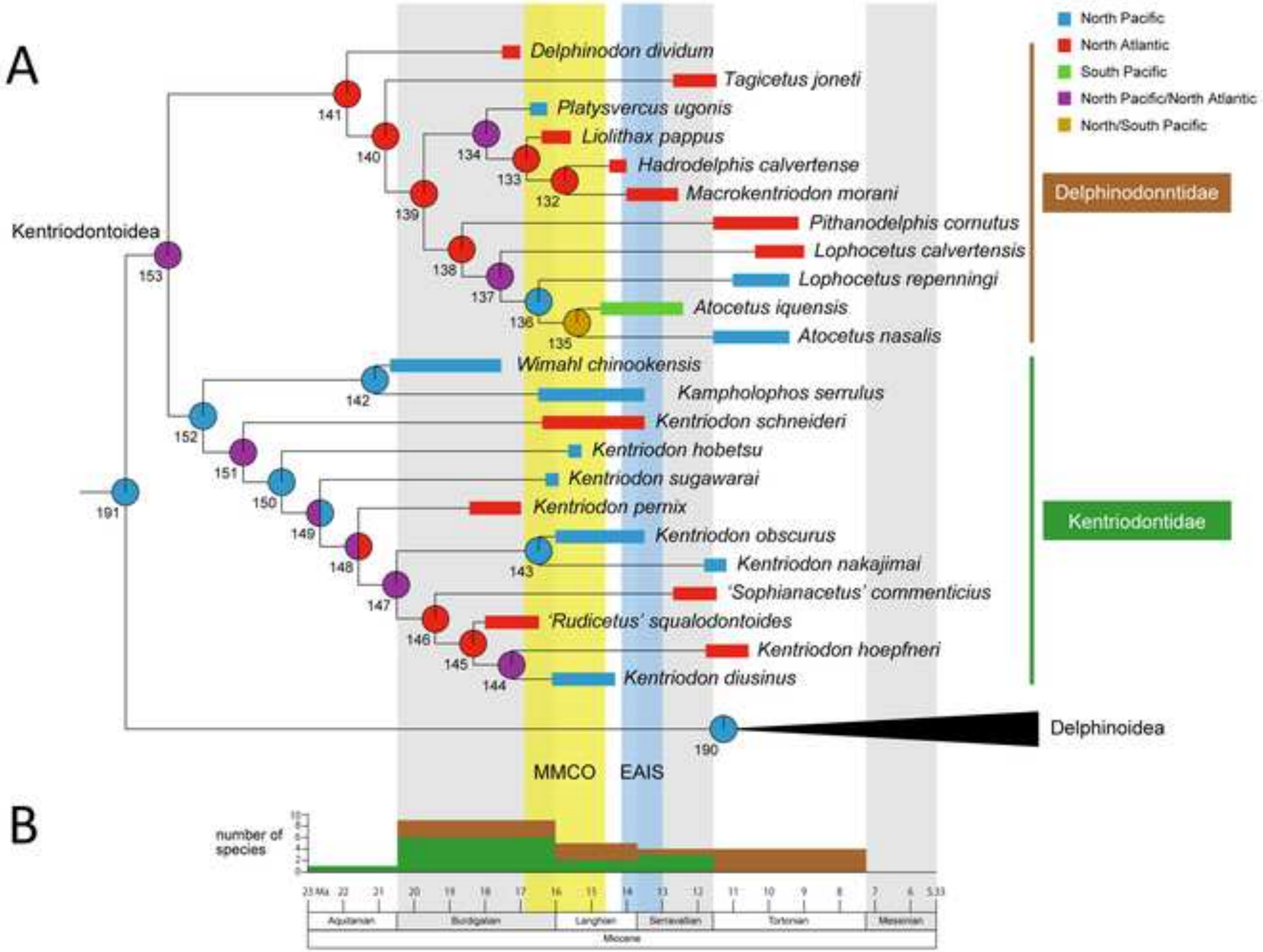

Click here to access/download
Supporting Information
S1 File.docx

Click here to access/download
Supporting Information
S2 File.nex

Supporting Information S3 File

Click here to access/download
Supporting Information
S3 File.tnt

Supporting Information S4 File

Click here to access/download
Supporting Information
S4 File.out

Click here to access/download
Supporting Information
S5 File.tre
