# Supplementary material for: An Early Miocene kentriodontoid (Cetacea: Odontoceti) from the western North Pacific, and its implications for their phylogeny and paleobiogeography
Source: PLoS One. 2023 Feb 15;18(2):e0280218. doi: 10.1371/journal.pone.0280218 (PMC9931143; doi:10.1371/journal.pone.0280218)
Supplement: Attachment — Submitted filename: GuoKohno_RebuttalLetter_220821.1.docx [file pone.0280218.s008.docx]

August 20, 2022

Dear Editors,

First of all, we deeply thank all the reviewers for their generous comments and suggestions for our manuscript on a new kentriodontid from Japan. We thoroughly revised the manuscript with additional data following recommendations and suggestions from the editor and reviewers. Then, our revised manuscript was greatly improved. Changes are recorded with the track and change function in the revised manuscript. We also submit the clean version of the revised manuscript after the tracked change copy. We followed almost all the editor’s and reviewers’ comments, which are written in *italicized blue*, and our responses are written in plane black below the editor’s and reviewers’ comments. We hope that our revision is acceptable for publication.

---------------

**Journal Requirements:**

*1. Please ensure that your manuscript meets PLOS ONE's style requirements, including those for file naming.*

We edited the manuscript followed by the style requirements.

*2. In your manuscript, please provide additional information regarding the specimens used in your study. Ensure that you have reported specimen numbers and complete repository information, including museum name and geographic location.*

We have reported specimen numbers and complete repository information, including museum name and geographic location.

*3. In your Data Availability statement, you have not specified where the minimal data set underlying the results described in your manuscript can be found. PLOS defines a study's minimal data set as the underlying data used to reach the conclusions drawn in the manuscript and any additional data required to replicate the reported study findings in their entirety. All PLOS journals require that the minimal data set be made fully available.*

We included all the minimal data set (especially for Fig. 7 and Fog. 8) in the Supporting Information files (S1 and S4 File, Figure S1-4, Table S1 and APPENDIX S5). We also described that we cited each minimal data set underlying the results in our revised manuscript.

*4. We note that Figure 1 in your submission contain map images which may be copyrighted. All PLOS content is published under the Creative Commons Attribution License (CC BY 4.0), which means that the manuscript, images, and Supporting Information files will be freely available online, and any third party is permitted to access, download, copy, distribute, and use these materials in any way, even commercially, with proper attribution. For these reasons, we cannot publish previously copyrighted maps or satellite images created using proprietary data, such as Google software (Google Maps, Street View, and Earth).*

The map of Figure 1 was extracted and modified from the Topographic Maps from The Geospatial Information Authority of Japan (GSI). The map from the GSI could be used for academic papers with CC BY 4.0 without permission. We added the information about the cited map data from GSI in the figure caption.

*5. Please take this opportunity to be sure you have met all of our guidelines for new species. For proper registration of a new zoological taxon, we require two specific statements to be included in your manuscript.*

We listed the LSID under the new clade names and inserted the sub-section "Nomenclatural Acts" into the Methods section.

**Review Comments to the Author**

**Reviewer #1**

*Your study makes 3 important contributions. It describes a new species of Kentriodontidae that has clear affinities with Liolithax and Lophocetus, you conduct a large phylogenetic analysis of odontocetes including most kentriodontidae, and you investigate the biogeographic history of this group. I have attached a copy of your ms with detailed comments and suggestions for improvements. Below are what I consider to be the most important issues to address:*

*1) Taxonomic revisions: I am supportive of naming new groups to further discussions on Kentriodontid phylogeny. However, I strongly urge you to define your clades as well as diagnose them. This is important because further studies will likely find slightly different relationships, and definitions will help future authors apply your names to their trees. Phylogenetic definitions are, in my opinion, most helpful, and Paul Sereno has published several papers how to define such clades so that they are stable.*

*You should also describe more of the existing literature concerning named taxa for clades within Kentriodontidae. For example de Muizon 1988 has a phylogeny of kentriodontids and uses the subfamilies Kentriodontinae, Lophocetinae, and Pithanodelphinae. You should discuss more how your results correspond to this earlier work.*

*Finally I think you need to explain more your reasoning on why a new superfamily is needed. Historically the clade you name Kentriodontoidea has been referred to as Kentriodontidae. In the ms you place kentriodontids in quotes, which usually indicates a paraphyletic assemblage. However your study actually supports its monophyly. I am assuming you did this because you are distinguishing the traditional definition from your new one. I would also encourage you to revise the diagnosis for Kentriodontidae in this paper, in addition to your new family Delphinodontidae.*

Agreed. First of all, we initially erected a new family name, the Delphinodontidae instead of elevation from the formerly established subfamily name to avoid the confusion of the definition and members to be included in the new family. But, following the suggestion from the reviewer, we replaced the newly proposed family name Delphinodontidae with the Lophocetidae that was previously established as a subfamily within the Kentriodontidae *sensu lato* (*s.l.*) and much earlier name for the clade that we erected in our study as a new family with the new definition. Earlier studies such as Barnes (1978, 1985) and Muizon (1988) assigned the whole *kentriodon*-like species as members of the monophyletic family Kentriodontidae, and established four subfamilies in the family such as the Kentriodontinae, Kampholophinae, Lophocetinae, and Pithanodelphinae. However, some recent studies (Lambert et al. 2017, Peredo et al. 2018, Kimura & Hasegawa 2019) suggested that the family Kentriodontidae *s.l.* was polyphyletic and that the Kentriodontinae or the Kentriodontidae *sensu stricto* (*s.s.*) only included the genera *Kentriodon, Rudicetus, Wimahl and Kampholophos*. Other genera as members of the Kentriodontidae *s.l.* were placed on the different monophyletic or polyphyletic groups independent from the Kentriodontidae *s.s*. Our study recognized these genera as an another monophyletic group and sister to the Kentriodontidae *s.s.*, meaning that the Kentriodontidae *s.l.* is also monophyletic, and thus, there should be an another family for these monophyletic clades. All the members of the Lophocetinae *sensu* Barnes (1978) and Pithanodelphinae *sensu* Barnes (1985) are included in the new subclade, while the Kampholophinae *sensu* Barnes (1978) is polyphyletic (*Kampholophos* is included in the Kentriodontidae *sensu stricto* and *Liolithax* is included in the new clade) in Lambert et al. (2017; 2018), Peredo et al. (2019) and in our analysis. In between the two subfamily names; i.e., the Lophocetinae Barnes, 1978 and Pithanodelphinae Barnes, 1985, the former was established much earlier within this newly recognized subclade to assign a new family name. We reflected the above discussion in our ms.

Also, our Kentriodontidae and Lophocetidae formed a monophyletic group, and the clade comprising the Kentriodontidae and the Lophodontidae was located as a sister to the superfamily Delphinoidea. In this context, this monophyletic group could be recognized as a same rank; i.e., superfamily, as well as the Delphinoidea. Thus, we elevated the Kentriodontidae *s.l.* to the superfamily. The diagnosis for the "new" Kentriodontidae (formerly the Kentriodontinae) has already been sufficiently discussed in previous studies (Predo et al., 2019; Guo and Kohno, 2021), and our result for the Kentriodontidae *s.s.* is identical to such previous studies, hence we did not repeat the diagnosis for the Kentriodontidae *s.s.*, instead we suggest it by the expression of the newly defined Kentriodontidae as "*sensu* Peredo et al. (2019)" as an authority for the new definition of the family name.

Finally, although there has been no question about the monophyletic relationship of the Kentriodontoidea in our study, it was often recognized as paraphyletic and/or polyphyletic. Accordingly, we had to distinguish our Kentriodontidae (*s.s.*) from the former usage of both the Kentriodontinae and the Kentriodontidae (*s.l.*). Accordingly, we put the quotation marks for the former usage of the Kentriodontidae to indicate the group that was previously recognized as paraphyletic and/or polyphyletic as well as to distinguish the traditional definition from our new one. We appreciate the reviewer’s careful suggestion and advice.

*2) In the methods section you need to detail how you conducted searches used to estimate Bremer support and bootstrap values.*

For the Bremer support, we estimated with the following settings: “Searching suboptimal trees, with score up to 30 worse than best (in 30 searches, each 1 worse than previous)”. Others are the default settings.

We edited the sentence in the method section with “To measure the stability of each node, we estimated Bremer support with score up to 30 worse than best (in 30 searches, each 1 worse than previous) and Standard Bootstrap support with 1000 replicates for the consensus tree”.

*3) I am not familiar with the program used for ancestral state reconstructions, but it seems important to describe how you (or the program) differentiates vicariance from dispersal. I am not saying that vicariance is impossible in the marine environment, but seems like dispersal might be more common.*

In S-DIVA, ancestral distributions in a phylogeny are reconstructed based on a three-dimensional cost matrix, in which extinctions and dispersals cost more than vicariance events. The biogeographic histories with the lowest cost are selected, while accounting for both phylogenetic and DIVA optimization uncertainty.

Our results recognized the common ancestor of the Kentriodontoidea spread out in the Northern Hemisphere (both North Pacific Ocean and North Atlantic Ocean). At the time of the first split of this ancestral population into two subgroups, one of which was left in the North Pacific Ocean and became the ancestor of the Kentriodontidae. Another one was left in the North Atlantic Ocean and later evolved into the Lophocetidae. For this event, the DIVA program can recognize that the common ancestor of the Kentriodontoidea was spread out in the Northern Hemisphere and did not migrate to a new area and form any one of the two families (dispersal), but recognize the later separation of the widely distributed population (vicariance), based on the framework of the phylogenetic relationships and distributions (or locality areas) of the whole members of the Kentriodontoidea in our analysis. We reflected these discussion more clearly in our ms.

*4) In the description it is unclear how your are using the term presphenoid and how it might relate to the ethmoid. I suggest you clarify your meaning and refer to Ichishima 2016 for the most recent discussion of the homology of bones in this region.*

According to Ichishima (2016), presphenoid is concealed by the cribriform plate; i.e., the ectethmoids and nasal septum. In UTHFM 00034, we can only clearly observe the nasal septum part of the presphenoid.

*5) In File S1 Fig. S2 looks corrupted, or maybe there are two trees, ones pasted over the other?*

We checked the figure in Fig. S2. There has only one figure and one tree. Maybe Fig. S2 collapsed with Fig. S3? We uploaded File S1 with a PDF version and hope to solve this issue.

**Other comments attached in the manuscript file:**

*l. 11, Rank is a separate issue from robustness. In other words a highly supported clade can be of low rank or of high rank.*

Agreed. We deleted the sentence “because of its robustness”.

*l. 17, The last sentence should be rephrased. It is unclear what two things are synchronous. Also unclear what vicissitudes refers to here.*

We edited the sentence with “The diversification and extinction of Kentriodontoidea could have been synchronously influenced by climate events during the middle Miocene”.

*l. 25, This may apply to genera but not to species with this group.*

Agreed. We replaced “species” with “genera” accordingly.

*l. 29, Just combine these two sentences and say some studies support kentriodontid monophyly and others do not, and cite examples of each,*

Agreed. We edited the sentence with “In particular, some of these studies [8,9] supported the monophyletic relationships of all previously known ‘kentriodontids’, which comprise the sister group of the Dephinoidea, while other studies [5–8] show quite different, polyphyletic topologies among them”.

*l. 40, Need to be clearer here. I assume you are referring to delphinoids replacing kentriodontids.*

Agreed. We edited the sentence with “In particular, the Delphinoidea originated from the late Miocene [12,13], and were replaced the kentriodontids afterward, while this turnover event also remains unexplained.”

*l. 50, Usually when one uses quotes here it is a sign that the group is not monophyletic and is invalid. However, I think your study support monophyly of Kentriodontidae, as historically used.*

Although there has been no question about the monophyletic relationship of the Kentriodontoidea in our study, it was often recognized as paraphyletic and/or polyphyletic. Accordingly, we had to distinguish our Kentriodontidae (*s.s.*) from the former usage of both the Kentriodontinae and the Kentriodontidae (*s.l.*). Accordingly, we put the quotation marks for the former usage of the Kentriodontidae to indicate the group that was previously recognized as paraphyletic and/or polyphyletic as well as to distinguish the traditional definition from our new one. Once again, we appreciate the reviewer’s careful suggestion and advice.

*l. 52, This is not clear to me. Do you mean determine its position in crown Odontoceti?*

Agreed. We edited the sentence with “Also, we determine its position in crown Odontoceti and re-examine ‘kentriodontid’ phylogeny with further species added.”

*l. 82, Need to describe how bootstrap and Bremer supports were determined. If Bremer support analyses not thorough enough, values can be inflated.*

For the Bremer support, we estimated with the following settings: “Searching suboptimal trees, with score up to 30 worse than best (in 30 searches, each 1 worse than previous)”. Others are the default settings.

We edited the sentence in the method section with “To measure the stability of each node, we estimated Bremer support with score up to 30 worse than best (in 30 searches, each 1 worse than previous) and Standard Bootstrap support with 1000 replicates for the consensus tree”.

*l. 93, Are you saying you used the consensus tree for this analysis? If so, it would be better to use a sample of the MP trees.*

For the tree we used for ancestral range reconstruction analysis, we used the ﬁnal tree (consensus tree) from the phylogenetic analysis. It was summarized from all the MP trees and has less conflict compared with a sample of the MP trees.

*l. 94, Is this text needed?*

Here we are explaining why we assigned distributional regions as follows.

*l. 119, A key question I have is why a new rank is needed.*

We recognized this monophyletic group as a superfamily because firstly this group is considered to be included two kentriodontid families (Kentriodontidae *sensu* Peredo et al., 2019 and our Lophodontidae as a family rank) based on our analysis and some of the recent studies such as Lambert et al. (2018) , Peredo et al. (2019), and Kimura and Hasegawa (2019), where "other species" that were formerly recognized as members of the Kentriodontidae *s.l.* were also recognized as another monophyletic subgroup. Second, in our analysis, the monophyletic group comprised of two monophyletic subgroups is located as sister to the superfamily Delphinoidea, and it is thought that it will be reasonable to recognize the group not be a family but could be a superfamily as same as its sister group, the superfamily Delphinoidea. In this case, the Kentriodontidae *sensu* Peredo et al. (2019) is reasonably included in the new superfamily Kentriodntoidea, and consequently, the new definition of an another monophyletic subgroup is also ranked as a family; i.e., the Lophodontidae.

*l. 139, If you are going to name a new family Delphinodontidae, I suggest you redefine and rediagnose Kentriodontidae as well*

The Kentriodontidae *s.s.* already had been redefined in recent studies (Peredo et al., 2019; Guo and Kohno, 2021). In our study, we obtained the same result for the clade Kentriodontidae *s.s.*; i.e., our analysis supported the results of Peredo et al. (2019) and Guo and Kohno (2021), and therefore, did not repeat the diagnosis for the "new" Kentriodontidae. Instead, we suggested it by the expression of the newly defined Kentriodontidae as "Kentriodontidae *sensu* Peredo et al. (2019)" as an authority for the new definition of the family name.

*l. 160, Should discuss Lophocetinae in this paper and how it differs from your Delphinodontidae.*

Once again, we appreciate the reviewer’s careful suggestion. We replaced the family name “Delphinodontidae” with a much earlier name “Lophocetidae” as elevated from subfamily to family, and we added some revisions for the “Lophocetidae” in the Discussion section. Please refer also to Review Comments (1)

*l. 206, This phrase is confusing*

We edited the sentence with “the premaxillary foramen located laterally from the midpoint to the premaxilla”.

*l. 234, This phrase is confusing as these structures are typically at different levels. Do you mean neurocranium?*

Agreed. We replaced “dorsoventral level of the supraoccipital” with “neurocranium”.

*l. 236, Are you sure it is not the other way around? The left condyle is higher than the right.*

Agreed. We edited the sentence with “…, and the posteroventral part of the cranium is higher on the left side than the right.”

*l. 238, foramen magnum? The midline would be between the condyes. Maybe you mean intercondyloid notch?*

Agreed. We edited the sentence with “…, while the vertex is slightly skewed right to the midline of the foramen magnum because of this deformation.”

*l. 244, check subject verb agreement in this section. Premaxillae is plural*

Agreed. We replaced “premaxillae” with “premaxilla” accordingly

*l. 252, unclear what this means*

We edited the sentence with “Medially, the premaxillae do not cover the external bony nares and the medial border of the premaxillae are oriented following the anterolateral wall of the external bony nares”.

*l. 257, How can the fossa be thickened? Do you mean the premaxilla itself.*

Agreed. We edited the sentence with “The premaxillary sac fossa is flat and gradually elevated posteriorly”.

*l. 296. This seems to contradict the previous sentence. Maybe keep this one and delete the previous sentence?*

Agreed. We edited the sentence with “Each alveolus is small and almost the same size (4–6 mm in transverse width) from the apex to the posterior part of the alveoli.”

*l. 314, This sounds not worth mentioning, unless you are stating they are unfused.*

Agreed. We deleted this sentence.

*l.317, Are the nasal septum and the presphenoid separate things? The homologies of the mesethmoid, cribriform plate are unclear. Should be clear what you mean here and maybe cite recent study by Ichishima 2016.*

According to Ichishima (2016), presphenoid is concealed by the cribriform plate, ectethmoids and nasal septum. In UTHFM 00034, we can only clearly observe the nasal septum part of the presphenoid.

*l. 343, Do they differ between the sides? If not leave this qualifier out.*

Agreed. We deleted “in the right frontal”.

*l. 353, This sentence is haed to follow and needs to be rewritten. Maybe subdivide into multiple sentences.*

Agreed. We subdivided this sentence into two sentences.

*l. 360, I do not think it is "almost" broken. Looks like only its base is preserved.*

Agreed. We deleted the word “almost”.

*l. 421, not really any more independent or separate than any other. I think you mean it is not the exclusive sister-group of another species.*

Agreed. UTHFM 00034 is included in the Lophocetidae. We edited the sentence with “UTHFM 00034 described here is included in the latter sub–clade and is located as a sister taxon to the *Macrokentriodon, Hadrodelphis*, and *Liolithax*.”

*l. 429, I think these were 1000 finds not replicates according to your methods section*

We set “Number of replications” to 1000 in the bootstrap method. We did not mention this in the methods section. We added this to the methods section.

*l. 441, This is a bit confusing. Do we know the ancestor for any of these clades?*

We have no described fossil record for the Delphinoidea until the late Miocene. We edited the sentence with “However, the ancestral taxon of the Delphinoidea is unknown until the late middle Miocene and is considered to have diversified just before the extinction of the kentriodontids *s.l.* based on fossil records.”

*l. 472, Need to discuss previous classification here, such as Muizon 1988 division into Kentriodontinae, Lophocetinae, and Pithanodelphinae.*

Agreed. We replaced the family name “Delphinodontidae” with “Lophocetidae” and added some revisions for the “Lophocetidae” to the Discussion section. Please refer to Review Comments (1)

l. 489, Should mention bootstrap and Bremer support values for this clade as well.

Agreed. We added the following sentence in the discussion section: “Also, bootstrap and Bremer support values (Fig. 7) indicated the stability of this phylogenetic relationship”.

**Reviewer #2**

*please excuse my tardy reply, and thank you very much for your patience in waiting for my review.*

*I have read over the above paper and support its publication on PLOS ONE after suitable moderate revisions.*

*Here you are my main comments:*

*1. Though mostly clear, the English text should be revised. I have included some possible edits in the attached revised manuscript file. That said, as I am not a native English speaker, I do not feel much qualified to help the authors on this very issue.*

We corrected all the grammatical mistakes and typographic errors, and then we edited the manuscript following the comments on the attached PDF file.

*2. (l. 231) Comparisons are completey absent from the Description section. Considering also that the diagnosis is not comparative, comparisons appear to be completely absent from the whole paper. This must be fixed before acceptance: detailed comparisons may be either interspersed within the description, or concentrated in a devoted paragraph at the end of the Systematics section. Anyway, including anatomical comparisons is mandatory in a paper that describes a new genus a species (otherwise, the legitimacy of the newly instituted taxon may even be disputed - in other words, it may not be obvious why Platysvercus ugonis should represent a new taxon).*

We added the Comparisons in the Discussion section, and included the details with anatomical comparisons to prove the new genus and species as a distinctive and definitive taxon.

*3. (l. 473) I am not sure whether splitting the kentriodontids into two families is a good idea. After all, there are recent phylogenies (e.g. those by Kimura & Hasegawa (2019) and Bianucci et al. (2022)) in which such clades are not recovered. Whereas there's more or less consensus on the existence of a group of kentriodontids "sensu stricto" including mostly Kentriodon species, the same cannot be said for the "delphinodontids". In addition, and closely related to the previous point, I wonder whether naming the new family after Delphinodon is a good idea. After all, Delphinodon is rather close to Kentriodon in morphology and size; furthermore, it is recovered as the basalmost representative of the "delphinodontids" in your analysis, as well as the earliest branching representative of Delphinida(!!) in the aforementioned papers by Kimura & Hasegawa (2019) and Bianucci et al. (2022).*

Agreed. We replaced the family name “Delphinodontidae” with a much earlier established name “Lophocetidae”. Earlier studies like Barnes (1978, 1985) and Muizon (1988) assigned the whole *kentriodon*-like species as the family Kentriodontidae, and included the subfamilies such as Kentriodontinae, Kampholophinae, Lophocetinae, and Pithanodelphinae. However, some recent studies (Lambert et al. 2017, Peredo et al. 2018, Kimura & Hasegawa 2019) suggested that the family Kentriodontidae only included the genera *Kentriodon, Rudicetus, Wimahl* and *Kampholophos*. We agree with the kentriodontids "*sensu stricto*" (*s.s.*) that is for this recent Kentriodontidae. Our study recognized another monophyletic group including all kentriodontid-like members excluding the taxa of the Kentriodontidae *s.s.*, ranked as same as and just sister with resent Kentriodontidae. Thus, there should have a family name for this new group to be assigned. All the members of the Lophocetinae and Pithanodelphinae are included in this group, while genera from Kampholophinae are spilt into each of these groups (*Kampholophos* is included in Kentriodontidae and *Liolithax* is included in the new group). To think both Lophocetinae Barnes, 1978 and Pithanodelphinae Barnes, 1985 are well-known clades, assigning a new family name from these terms is more stable and helpful to future work. We choose Lophocetidae for the name of this new group because Lophocetinae Barnes, 1978 is the oldest name and has priority. We added these sentences to the Discussion section.

*4. (l. 137) If you decide for keeping the new family Delphinodontidae, redefining the family Kentriodontidae with a new diagnosis would be very helpful here, and much in line with the "spirit" of the Principle of Coordination.*

The Kentriodontidae already had been redefined in a recent paper (Guo and Kohno, 2021). In this paper, we obtained the same result for the clade Kentriodontidae, our analysis supported the results of Guo and Kohno.

Guo Z, Kohno N. A new kentriodontid (Cetacea: Odontoceti) from the early to middle Miocene of the western North Pacific and a revision of kentriodontid phylogeny. PeerJ. 2021;9: e10945. doi:10.7717/peerj.10945.

*5. (l. 523) I think that the palaeobiogeographic paragraph of the Discussion ignores the occurrence of Kentriodon sp. in the Lower Miocene of Peru. Please discuss the data on Kentriodon presented in the papers by Bianucci et al. (2018) and Di Celma et al. (2019). In addition, the Middle and Late Miocene of the Pisco Formation of Peru includes many kentriodontid-like specimens (Collareta et al., 2021).*

The paleobiogeographic reconstruction is highly dependent on the topology of taxa based on the phylogenetic analysis. However, there have some difficult points that many nicely preserved specimens such as those discovered in Peru, have yet been undescribed. Others like many specimens have been poorly preserved, we could not reconstruct their phylogenetic relationships. Our study includes all available kentriodontid taxa than any previous papers. We believe our result could be reliable at the current stage. We added a sentence to explain this issue: “Further analysis could be improved current reconstruction when even more specimens could be added to the cladogram. Recently, many specimens including kentriodontids had been reported such as those undescribed specimens discovered in Peru [Collareta et al. 2021, Bianucci & Collareta 2022]”.

*6. (l. 574) A more general reflection: the fossil record is certainly intrinsically fragmentary... However, Bianucci et al.'s phylogeny (in the supp. mat.), in which several kentriodontid-like dolphins (including many "delphinodontids") are more derived than (and not sister group to) kentriodontids s.s. avoids the need for a ghost lineage of basal delphinoids ranging from the Aquitanian/Burdigalian transition through the Burdigalian and Middle Miocene well into the Tortonian. I think you should consider (=mention and discuss) the alternative phylogenetic reconstruction by the recent paper by Bianucci et al. (2022).*

Bianucci et al. (2022) used the data matrix with 148 taxa (18 kentriodontid taxa) and 374 characters, which was modified from Boessenecker et al. (2020) that originated from Lambert et al. (2017). They've modified several characters and codings and added several taxa including kentriodontids such as *Kentriodon diusinus*, *K. obscurus*, *K. schneideri*, and *Wimahl chinookensi*. Our data matrix has 107 taxa (21 kentriodontid taxa) and 387 characters and covers all the taxa operated in Bianucci et al. (2022) only without *Kentriodon diusinus* because of the extremely low coding proportion. When we submitted the manuscript in October 2021, Bianucci et al. (2022) paper had not been published. However, for the phylogenetic relationship of kentriodontids, the topology of Bianucci et al. (2022) is just like Lambert et al. (2017). Because our studies (e.g., Guo and Kohno, 2021 and this study) have discussed the result of the phylogenetic relationship of the kentriodontids in Lambert et al. (2017), and thus, we also cited Bianucci et al. (2022) with Lambert et al. (2017) in our ms.

**Other comments attached in the manuscript file:**

*l. 25, Awkward sentence... please try and rephrase*

We edited the sentence and split it into two sentences with “Although many studies have concentrated to understand their inter–generic and inter–specific relationships within the Delphinida, some of the previously described specimens of ‘kentriodontids’ were fragmentary, and they have sometimes been classified as paraphyletic and/or unresolved taxa.”

*l. 122, Phraseology may be improved in this paragraph. I suggest a simpler and more schematic approach (e.g. avoid definite articles where possible).*

*Also in l. 205, Here again, putting the diagnosis in a more schematic writing style may be helpful.*

We cited the diagnostic characters based on the result of our phylogenetic analysis. We think keeping the style of description of each character states are easier to understand than rewriting it to a simpler style.

*l. 149, This condition is also observed in some Kentriodon species, isn't it?(cf. internasal fossa; see also Muizon, 1991)*

The deep cleft between the nasals (character 134) cannot be seen in the *Kentriodon* species included in our cladogram. The internasal fossa is another character (character 131) independent from character 134. The internasal fossa (character 131) can be seen in some kentriodontids like *Tagicetus joneti* and *Atocetus nasalis*, but not in *Kentriodon* species.

*Table. 1, Minimum?*

About the terminology of this measurement, “Minor diameter” is more precise.

Kemper, Catherine. (2004). Osteological variation and taxonomic affinities of bottlenose dolphins, Tursiops spp., from South Australia. Australian Journal of Zoology - AUST J ZOOL. 52. 10.1071/ZO03026.

*l. 218, Early (Lower), Middle and Late (Upper) Miocene are now formal units.*

According to the International Commission on Stratigraphy, early Miocene is a sub-epoch that is comprised of two official stages: the Aquitanian and Burdigalian, and the sub-epoch is not an official subdivision for the Epoch based on the International Chronostratigraphic Chart as of 2022/02. In this regard, we've retained the sub-epochs; i.e., early (lower), middle and late (upper) as lower cases. However, if our understanding is incorrect, we have to revise all of them.

*l. 231, Please, include frequent references to the figures in the descriptive sections of the paper.*

We added some references to the figures in the Description section.

*l. 251, ?? Not clear to me. How could the bony nares be covered by some other bone?*

In some Delphinoidea, the premaxilla is covered or roofed the part of the bony nares anterolaterally in dorsal view, and the anterolateral wall of the bony nares cannot be observed. On UTHFM 00034, the premaxilla is not covering the bony nares. We edited the sentence with “Medially, the premaxillae do not cover the external bony nares and the medial border of the premaxillae are oriented following the anterolateral wall of the external bony nares.”

*l. 259, It appears as more wedge-shaped in Fig. 2b*

Agreed. We replaced the word “knob–like” with “wedge-shaped” accordingly

*l. 277, This is quite a vague indication; maybe to be better specified?*

Agreed. The prenarial triangle on the premaxilla is a wide area. We replaced “prenarial triangle” with “premaxillary foramen” accordingly.

*l. 321, What's this measurement?*

This measurement is the anteroposterior length of the keel of the presphenoid. We replaced “11 mm” with “11 mm in anteroposterior length” accordingly.

*l. 330, In the diagnosis of Delphinodontidae, you listed the presence of a deep cleft at the nasal-nasal suture as a diagnostic character of Delphinodontidae*

Agreed. A deep cleft at the nasal-nasal suture exists on UTHFM 00034. This sentence we described here is to indicate the internasal fossa is not present on UTHFM 00034. The internasal fossa can be seen in some kentriodontids like *Tagicetus joneti* and *Atocetus nasalis*.

*l. 339, Unclear: what's the subject of this sentence?*

The suture between the left and right frontal is a morphological character of Odontoceti. Some odontocetes have no suture between the left and right frontal, while some have deep crests.

*l. 342, ??*

The orbit is dorsoventrally located just as same as with the lateral edge of the rostrum. We added the word “dorsoventrally” in the sentence.

*l. 346, What direction for the optic canal?*

The optic canal is opened dorsolaterally. We edited the sentence with “It is located at the level just anterior to the choana and opened dorsolaterally.”

*l. 353, This sentence is long and a bit tortuous. I would suggest some rephrasing - maybe two shorter sentences may prove clearer?*

Agreed. We split this sentence into two sentences.

*l. 370, ?? I don't think that's the right term here.*

Agreed. We edited the sentence with “The post–tympanic process is slightly concaved.”

*l. 378, I think that's all included in the meaning of "fused" (when applied to different bones).*

Agreed. We deleted “but the sutures are unclear.”.

*l. 386, To be, possibly, better clarified.*

Agreed. Because the nuchal crest is higher than the maxilla, so the dorsal profile is unrelated to the anterior maxilla. We already mentioned this above, so we deleted the sentence “the shape is not related to the dorsal shape of the maxilla immediately anterior to it.”

*l. 399, What is unclear? The profile of the condyloid process, or even whether it's present or not?*

The dorsal condyloid fossa is originally placed just dorsal to the condyle. However, in UTHFM 00034, both areas just above both condyles are broken. So, the detail of the dorsal condyloid fossa is unclear.

*l. 399, Not indicated in Fig. 5B*

The pedicle of the condyle can be observed in ventral view. We indicated the pedicle of the condyle in Fig. 4B.

*l. 400, Not so clear from the figure...*

The intercondyloid notch is originally depressed anteriorly. The U–shaped intercondyloid notch can be observed in the ventral view (Fig. 4).

*l. 421, Please be clear in identifying the two "sub-clades" as Kentriodontidae and Delphinodontidae (unless you decide to abstain from naming the new family Delphinodontidae). Mentioning the 'kentriodontids', in inverted commas, versus the kentriodontids "sensu stricto" does not help much the reader. Alternatively, you may talk about "kentriodontid-like dolphins" to refer to the 'kentriodontids'.*

Agreed. We changed the word “'kentriodontids'” to "kentriodontid-like dolphins".

*l. 519, ??*

We edited the sentence with “…but each kentriodontoid family cannot be defined by any unrivaled autapomorphic characters.”

*l. 527, Unclear sentence*

We edited the sentence with “This initial diversification is interpreted to be the range expansion of their distribution.”

*l. 541, The following "odontocete" reference may prove relevant to cite here:*

*Peri et al. (2019).- An Inticetus-like (Cetacea: Odontoceti) postcanine tooth from the Pietra leccese (Miocene, southeastern Italy) and its palaeobiogeographical implications.- Neues Jahrbuch für Geologie und Paläontologie, Abhandlungen, vol. 291, p. 221-228.*

Thank you for the information. We cited this paper below the sentence.
