# Supplementary material for: An Early Miocene kentriodontoid (Cetacea: Odontoceti) from the western North Pacific, and its implications for their phylogeny and paleobiogeography
Source: PLoS One. 2023 Feb 15;18(2):e0280218. doi: 10.1371/journal.pone.0280218 (PMC9931143; doi:10.1371/journal.pone.0280218)
Supplement: Attachment — Submitted filename: GuoKohno_RebuttalLetter_221217.docx [file pone.0280218.s009.docx]

December 17, 2022

Dear Editors and reviewers,

We thank again the editor and reviewers for their generous comments and suggestions for our manuscript on a new kentriodontoid from the Lower Miocene of Japan. The reviewers’ comments and suggestions greatly improved our manuscript. In spite of their constructive reviews, we have to apologize for the delay to revise our manuscript because of the current situation of the novel coronavirus crisis as an eighth wave in November and December. Although delayed, we revised the manuscript thoroughly and improved it with additional data following recommendations and suggestions from reviewers. Changes are recorded with the track-and-change function in the revised manuscript. We followed most of the reviewers’ comments. Reviewers’ comments are written in blue below. Our responses are written in black.

-=-=-=-=-=-=-=-=-=-=-=-=-=-=

Reviewer #2 (Alberto Collareta):

Dear Editors and Authors,

I have read over the revised version of the paper by colleagues Guo & Kohno (and especially their responses to the reviewers' comments). It is clear that most of the reviewers' comments have been carefully handled by the authors, resulting in a strengthened manuscript.

1. The authors have addressed my previous comment on the need for detailed osteoanatomic comparisons by adding a paragraph entitled “Inter-specific comparisons in the Lophodontidae” to the Discussion. Considering the final sentence of this paragraph (“Based on our phylogenetic analysis with species level comparisons above, we propose a new genus and species Platysvercus ugonis for UTHFM 00034 within the Lophocetidae”), the whole paragraph may rather be placed within the Results. Such a change may need to be accompanied by some local rewriting.

Agreed. We changed the location of the paragraph “Inter-specific comparisons” to the place just before the Results. And changed the location of the final sentence in the Results.

2. I understand why the authors are reluctant to redefine Kentriodontidae. However, as also suggested by the other reviewer, recalling the diagnosis of this family (as retrieved in the present work, and sensu Peredo) would surely prove useful.

We appreciate the reviewer’s advice. Although our result of the diagnosis of Kentriodontidae was completely the same one as was of Guo & Kohno (2021) and slightly different from that of Peredo et al. (2018). We were initially afraid that if we recalled the diagnosis extracted from Guo & Kohno (2021), it would result in a high text overlapping. We were initially afraid that this would exceed the limit from the terms of the journal. Regarding this, we did rewrite the diagnosis based on that of Guo & Kohno (2021) in our manuscript.

3. My main concern with this manuscript still regards the English text. Though the majority of the manuscript is clear, not all the additions to the text have been ameliorative of this aspect. However, I am not a native English speaker, and as such, I am not particularly qualified to comment on this very issue.

We corrected the English text as much as possible. We also got professional English proofread before we resubmitted our manuscript to this journal. We hope our manuscript is acceptable for publication.

Reviewer #3 (Gabriel Aguirre-Fernández):

Dear authors,

I have been assigned to review this manuscript without being involved in the first round of reviews (of which I had access, along with your replies). The manuscript is sound and contains important new information regarding a new species of kentriodontid. The taxon sampling is great (it includes many kentriodontids) and the phylogenetic analysis is adequate. I have never performed a biogeographic analysis as done here using S-DIVA and therefore my expertise falls out of it, but I would like to share some general concerns that I hope are relevant and helpful below.

General comments:

Taxonomy

I understand the basis for suggesting a new rank for the group traditionally known as Kentriodontidae, namely the finding in this study of two subclades: (A) containing Kentriodon and related taxa (in the manuscript referred to “Kentriodontidae” or “Kentriodontidae sensu stricto”); and (B) a subclade containing taxa originally assigned to Kentriodontinae (most notably, Delphinodon dividum) together with taxa assigned to the traditionally-known subfamilies Lophocetinae and Pithanodelphinae (in this version of the manuscript collectively referred to as Lophocetidae). The assemblage of taxa in subclade B does not allow the use of the terms Lophocetinae and Pithanodelphinae in the traditional sense anymore.

Despite this, I think that the use of new rankings (Kentriodontoidea, and within this clade Kentriodontidae and Lophocetidae) does not help and in fact brings more confusion. A good example is in this manuscript: to differentiate between the “old” (traditional) term Kentriodontidae and the new term Kentriodontidae, the authors chose to refer to the old term Kentriodontidae using quotation marks and the informal name (“kentriodontids”) in the abstract and introduction, but this generally implies the group is paraphyletic or polyphyletic (as mentioned in the previous round of reviews). In this manuscript, it means indeed a monophyletic grouping that is used as a placeholder for what later would be defined as Kentriodontoidea. In the discussion we also find a new term “Kentriodontidae sensu lato” that somewhat is a synonym of the “kentriodontids” of the abstract and introduction and Kentriodontoidea of the title, the systematic section, and some other parts of the text. The use of so many names for the same clade throughout the manuscript is confusing and, in my view, does not help to better understand the message nor helps future papers probably dealing with similar issues.

I strongly encourage you to keep the traditional definition of family Kentriodontidae and to avoid any re-rankings. The subclades can be referred to using letters (as I did in the previous paragraph) or alternatively as the kentriodontinae and the non-kentriodontinae clade; both provide a quick aid to navigate through the information and avoid adding to the already convoluted taxonomy of this group.

I appreciate the reviewer's advice. The family Kentriodontidae sensu lato was defined by Barnes (1978), and the Kentriodontidae included all the previously known kentriodontid taxa as a monophyletic group. However, some recent papers (Lambert et.al, 2017; Peredo et.al, 2018; Kimura & Hasegawa, 2019) analyzed the phylogeny of the kentriodontid taxa again and proposed different results that *Kampholophos,* *Kentriodon* and *Rudicetus* are forming a monophyletic group (group A), but other kentriodontid taxa were recognized as paraphyletic or polyphyletic. Thus, Peredo et.al (2018) redefined the Kentriodontidae that it included only the taxa which were recognized as monophyletic (group A). This paper also shows the same result with this group, but most importantly the other taxa previously recognized as paraphyletic, kentriodontids sensu lato were also recognized as monophyletic as was also by Guo and Kohno (2021) and consisted of another subclade within the monophyletic Kentriodontidae sensu lato. So we followed the recent definition for the family Kentriodontidae by Peredo et al. (2018) and Guo and Kohno (2021) as the Kentriodontidae sensu stricto (i.e., group A).

For the taxa other than Kentriodontidae, such as *Atocetus*, *Delphinodon*, *Hadrodelphis*, *Lophocetus* and *Liolithax*, the phylogenetic relationships of these taxa were controversial in the context of phylogenetic relationships among recent studies (e.g., Lambert et.al, 2017; Peredo et.al, 2018; Kimura & Hasegawa, 2019; Guo & Kohno, 2021). We did a phylogenetic analysis based on the character listings for the data matrix of Guo & Kohno (2021), and our result showed that such taxa other than Kentriodontidae sensu stricto, also formed a monophyletic group (group B) as has also been recognized by Guo and Kohno (2021). In addition, it showed a same degree of taxonomic diversity with sufficient branch robustness like that of the Kentriodontidae sensu stricto (group A). In this regard, another monophyletic group (group B) should also be recognized as the same rank as the family Kentriodontidae sensu stricto (group A). If we keep the traditional rank of Kentriodontidae for the group A plus group B, there also should be assigned a name “Lophocetinae" (group B) for the kentriodontids except the traditional rank of the Kentriodontinae (group A) with a different definition from the previous studies. We believe it will also not help in future works and will not be able to avoid similar confusions that were suggested by the reviewer. Although earlier studies such as Barnes (1978, 1985) and Muizon (1988) established four subfamilies for kentriodontids such as Kentriodontinae, Kampholophinae, Lophocetinae, and Pithanodelphinae, only the members of the Lophocetinae sensu Barnes (1978) and Pithanodelphinae sensu Barnes (1985) were included in our new subclade (group B). So we assigned this newly recognized subclade (group B) as the Lophocetidae because the name Lophocetinae Barnes, 1978 was established much earlier among the names for the taxa included in our subclade. We also considered using a new name such as the Delphinodontidae at first, because *Delphinodon dividum* is the first described species in group B, however, like the comments from Reviewer #1 in the first round of the revision, a crown taxon name like Lophocetinae or Pithanodelphinae will more help to understand previous and future works.

Also, Kentriodontidae (group A) and Lophocetidae (group B) formed a monophyletic group. Due to it having almost the same degree of taxonomic diversity (21 species in our analysis, actually at least 30 species in the record) as Delphinoidea (37 modern species), and having numerous synapomorphies (12), it has almost the same robustness as the superfamily Delphinoidea. Also, bootstrap and Bremer support values indicated sufficient stabilities for this phylogenetic relationship. In this regard, this monophyletic clade (group A+B) must be recognized as a clade of superfamilial rank as is the case for their sister taxon, the Delphinoidea. Hence, we propose the Superfamily Kentriodontoidea, to indicate the same diversity and robustness as the Delphinoidea.

We also appreciate the reviewer’s advice regarding the confusion of the taxonomic names, we replaced the term “Kentriodontidae sensu lato” with “kentriodontids sensu lato” indicating all the kentriodontid taxa to avoid such confusion.

Biogeography

Insights into chronology and distribution of kentriodontids are most needed, particularly because they may help to disentangle their complex taxonomic history, which is far from settled. I particularly like the part of the discussion where climate change and faunal turnovers are put into context. I think that the methodology section is missing some relevant details: in the methods the data was apparently assembled into 7 regions (A–G), but in Figure 8 there are some new categories: North Pacific/North Atlantic and North/South Pacific; in supplement 1, Figure S4 I see almost 40 categories depicted. I understand these are ranges, and some cetaceans are cosmopolitan and others have a very restricted distribution, however, I do not understand the need of pie charts and the reporting of probabilities in the results then, because almost all the pies are uniform in color and the probabilities reported are frequently 100%. Refer for example to Figure 8: the clade that includes the new taxon Platysvercus ugonis is phylogenetically reconstructed along with Liolithax, Hadrodelphis and Macrokentriodon (all 3 from the North Atlantic), further, Delphinodon and Tagicetus (the closest stemward relatives) are also from the North Atlantic. If the purple category (North Pacific/North Atlantic) did not exist, I would expect the pie in node 134 to show ¾ of red (indicating that the origin was most likely in the North Atlantic and Platysvercus is an immigrant in the North Pacific (there are probably more complex ways of seeing this, with varying percentages). A very simplistic a discrete character state reconstruction (reconstructing distribution as a state under parsimony) would again probably place a 100% red color in the node 134. In short: I do not understand why there are categories that lump areas (as the purple and yellow-brown categories in Figure 8), they seem counterintuitive to me, but again, I cannot really bring more constructive comments here.

The categories such as AB is indicating the biogeographic region distributed in both regions A and B. For instance, we input the biogeographic regions ABCDEFG for the extant species *Orcinus orca* (because it is distributed globally). The biogeographic regions for them were recognized as the change between the ancestral node to the descendant from region A to region AB indicating a dispersal event.

About the percentage at the node, according to Yan et.al (2004), “…the RASP version of DEC exports the likelihood of all possible biogeographic scenarios estimated at a given node. Akaike weights (AICw) for alternative models can then be calculated and interpreted as the relative probability of different ancestral ranges, which are displayed as pie charts on the nodes of a phylogenetic tree (Beaulieu et al., 2013). Thus, the additions to the DEC model in RASP allow for a more comprehensive evaluation of the degree of ancestral state uncertainty in biogeographic reconstructions.” The percentage at the node could normally occur even in all the phylogenetic trees showing one pattern in kentriodontids (our kentriodontoids) according to this algorithm.

Specific comments, number referring to line number in the clean copy (the one lacking track changes):

94-which consensus tree, majority rule or strict?

Strict. In this paper, we only use one strict tree for the phylogenetic result. We use this strict tree as a ‘user–specified consensus tree’ in the S–DIVA, to plot the ancestral distribution results. We added the sentence with “…, the strict consensus tree from the phylogenetic analysis”

Table 1- check major and minor diameter of temporal fossa proper, are the values swapped?

No. We follow the measurements in Perrin (1975). The major diameter of temporal fossa proper is indicated as the height of the temporal fossa viewed from posterodorsally, while the minor is indicated as the mediolateral length of the temporal fossa. UTHFM 00034 has a low but wide temporal fossa.

Perrin, W. F. 1975. Variation of spotted and spinner porpoise (genus Stenella) in the eastern Pacific and Hawaii. Bulletin of the Scripps Institution of Oceanography of the University of California 21:1–206

451-and Lipotidae?

Corrected.

452-node 192 not shown in Figure 8, maybe remove from the main results as it is shown only in supplement?

We did not include node 192 in Figure 8 just because we wanted to make this figure compact. We think the node 192 is also a piece of important information so we edited the sentence in line 452 with “(node 192, A, probability = 100%, shown in Supporting Information S1)”.

601&602-strange wording, maybe “could improve the current…”?

Agreed. We edited the sentence with “could improve the current”.

634- either these turnover events or this turnover event

Corrected. We edited the sentence with “this turnover event”.

644- …of the kentriodontids were refined

Corrected.

657- the kentriodontids (no caps) decreased… in diversity?

Corrected.

Reference 51 – Capitalize Pisco Formation, Peru, and Humboldt

Corrected.

Figure 2B-“preorbital process” is showing the postorbital process; there is a missing arrow for the supraoccipital; arrow pointing the posterolateral sulcus is a few mm too short and not showing the sulcus; the “presphenoid” is in my interpretation the ethmoid.

Corrected. According to Ichishima (2016), presphenoid is concealed by the cribriform plate, ectethmoids and nasal septum. In UTHFM 00034, we can only clearly observe the nasal septum part of the presphenoid.

Figure 3B, Figure 4B (and description of supraoccipital)- the “pedicle of occipital condyle” is a term I have never seen before and it is not in Mead and Fordyce’s (2009) lexicon. Is it relevant to have? Could you provide a reference on who defined that term?

The term “pedicle of occipital condyle” is from the character #231. Also in Tanaka and Fordyce, (2014, 2015) character #284; Tanaka et al. (2017) character #166. Some fossil taxa such as kentriodontids, Platanistoidea and some early Odontoceti preserved this feature.

Figure 4B- I interpret the “posterior lamina of pterygoid” as the medial lamina of the pterygoid of the pharyngeal crest of Mead and Fordyce (2009).

According to Mead and Fordyce (2009), “The medial lamina forms the ventrolateral wall of the nasal passage and part of the attachment of the palatal sphincter and carries the eustachian notch”. Also, as shown in their Figure 21c, the medial lamina is located just above the pterygoid hamulus. Also, in Mead and Fordyce “In *Tursiops* each (pharyngeal) crest is a continuous structure formed by the basioccipital crest posteriorly and the medial lamina of the pterygoid anteriorly.” Thus, the pharyngeal crest is not only formed by the medial lamina. Also, in Mead and Fordyce “The choanae in odontocetes consist of the posterior borders of the pterygoids and the vomer”. Accordingly, the pharyngeal crest located in the choanae of UTHFM 00034 should be formed by the posterior lamina of pterygoid.

------=-=-=-=-=-=-=-=-=-=-=

Once again, thank you for your consideration.

Sincerely,

Zixuan Guo

Department of Life and Environmental Sciences, University of Tsukuba

guo_z@geol.tsukuba.ac.jp

Naoki Kohno

Department of Geology and Paleontology, National Museum of Nature and Science,

Department of Life and Environmental Sciences, University of Tsukuba

Kohno@kahaku.go.jp
